# Southern introgression increases adaptive immune gene variability in northern range margin populations of Fire‐bellied toad

**DOI:** 10.1002/ece3.7805

**Published:** 2021-06-27

**Authors:** Binia De Cahsan, Katrin Kiemel, Michael V. Westbury, Maike Lauritsen, Marijke Autenrieth, Günter Gollmann, Silke Schweiger, Marika Stenberg, Per Nyström, Hauke Drews, Ralph Tiedemann

**Affiliations:** ^1^ Unit of Evolutionary Biology/Systematic Zoology Institute of Biochemistry and Biology University of Potsdam Potsdam Germany; ^2^ GLOBE Institute University of Copenhagen Copenhagen Denmark; ^3^ Department of Evolutionary Biology University of Vienna Vienna Austria; ^4^ Herpetological Collection Natural History Museum Vienna Vienna Austria; ^5^ Ekoll AB Malmö Sweden; ^6^ Stiftung Naturschutz Schleswig‐Holstein Molfsee Germany

**Keywords:** *Bombina bombina*, heat shock protein, introgression, major histocompatibility complex, scaled mass index

## Abstract

Northern range margin populations of the European fire‐bellied toad (*Bombina bombina*) have rapidly declined during recent decades. Extensive agricultural land use has fragmented the landscape, leading to habitat disruption and loss, as well as eutrophication of ponds. In Northern Germany (Schleswig‐Holstein) and Southern Sweden (Skåne), this population decline resulted in decreased gene flow from surrounding populations, low genetic diversity, and a putative reduction in adaptive potential, leaving populations vulnerable to future environmental and climatic changes. Previous studies using mitochondrial control region and nuclear transcriptome‐wide SNP data detected introgressive hybridization in multiple northern *B. bombina* populations after unreported release of toads from Austria. Here, we determine the impact of this introgression by comparing the body conditions (proxy for fitness) of introgressed and nonintrogressed populations and the genetic consequences in two candidate genes for putative local adaptation (the MHC II gene as part of the adaptive immune system and the stress response gene HSP70 kDa). We detected regional differences in body condition and observed significantly elevated levels of within individual MHC allele counts in introgressed Swedish populations, associated with a tendency toward higher body weight, relative to regional nonintrogressed populations. These differences were not observed among introgressed and nonintrogressed German populations. Genetic diversity in both MHC and HSP was generally lower in northern than Austrian populations. Our study sheds light on the potential benefits of translocations of more distantly related conspecifics as a means to increase adaptive genetic variability and fitness of genetically depauperate range margin populations without distortion of local adaptation.

## Graphical abstract

Endangered Northern edge populations (Northern Germany, Sweden) of the fire‐bellied toad were introgressed by Southern specimens from Austria. Introgressed Swedish populations were elevated in body weight and allelic diversity of an adaptive immune gene (MHCII), while diversity at a heat shock protein gene stayed unaffected. This points towards a benefit of edge populations from long‐distance translocation.

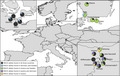

## 1 INTRODUCTION

A potential negative consequence of genetic admixture between differentiated gene pools is a reduced fitness due to distortion of local adaptation, in particular in the F1 generation (Allendorf et al., 2001; Brideau et al., 2006; Rhymer & Simberloff, 1996; Willett, 2006). However, intra‐ and interspecific genetic exchange could also provide beneficial alleles and subsequently promote the acquisition of adaptive traits (Hedrick, 2013). This has been seen in previous studies on wild populations of salamanders, mice, and sunflowers (Anderson et al., 2009; Arnold & Kunte, 2017; Fitzpatrick & Shaffer, 2007; Lexer et al., 2003; Whitney et al., 2006, 2010). These studies have all shown that introgressive hybridization between divergent gene pools can lead to positive fitness effects. This form of introgression could be particularly beneficial for organisms living in an environment prone to future anthropogenic and climatic changes (Arnold et al., 2016; Arnold & Kunte, 2017; Janes & Hamilton, 2017).

Many conservation genetics studies on endangered species assess variation at presumably neutral molecular markers (e.g., microsatellites, mitochondrial DNA; McCartney‐Melstad & Shaffer, 2015; Oswald et al., 2017; Schröder et al., 2012). These markers are useful to infer the demographic state of a population and may provide estimates for population history (bottlenecks, founder effects), relatedness, effective population size, and connectivity among populations (Hofman et al., 2007; Jehle & Arntzen, 2002). However, these loci may not reflect adaptive processes within and between populations. To identify consequences of small population sizes for adaptation, it is preferable to concentrate on genes under selection, in particular those under positive selection where genetic variation itself may be adaptive and/or where local adaptation may have favored locally different alleles. Commonly studied examples of such genes are Major Histocompatibility Complex (MHC) (e.g., Sommer, 2005) and Heat Shock Protein (HSP) genes (Robert et al., 2001; Sørensen et al., 2009). An increased variability of the MHC locus has been shown to correlate with an increase in population size (Madsen et al., 1999).

Major histocompatibility complex class I and II genes play an essential role in the adaptive immune response of vertebrates (Benacerraf, 1981; Hedrick, 2013; Klein, 1986; Mak & Saunders, 2006). This gene family is known to be associated with population viability and individual reproductive success (Manlik et al., 2019; Sepil et al., 2013). The genes encode cell‐surface glycoproteins that bind and present antigenic peptides to T cells, initiating an immune response. In exons involved in encoding parts of the antigen‐binding region, a higher ratio of nonsynonymous over synonymous substitutions is often observed (Piertney & Oliver, 2006). This excess of nonsynonymous substitutions, inferred as adaptive variation, is thought to be maintained by balancing selection, where heterozygosity is an advantage in response to pathogens and parasites (Hedrick, 2002). MHC genes also play a role in disassortative mating preferences, where females choose males based on their MHC gene diversity to avoid inbreeding or gain adaptive variants (Milinski, 2006; Potts et al., 1994). Therefore, MHC represents a suitable marker to assess post‐translocation fitness consequences in small, inbred populations (Madsen et al., 1999).

Heat shock proteins (HSPs) first became known for their ability to prevent the aggregation of proteins and assist in the refolding of misfolded proteins under stress conditions. HSPs are molecular chaperons which have been repeatedly discussed as important players in the adaptation to various perturbations of the environment, most notably heat stress (see Sørensen et al., 2003 for review). Here, in particular the expression of HSPs is altered to cope with environmental change (Sørensen et al., 2003), which has also been evidenced in amphibians (Heikkila, 2010). In tadpoles of *Rana*, a correlation between heat tolerance and HSP gene expression could be established (Sørensen et al., 2009). However, HSPs are also essential during a number of other processes under normal conditions (Bukau et al., 2006; Bukau & Horwich, 1998). At least in mammals, HSPs are considered important also during adaptive immune responses of the organism (Srivastava, 2002).

The European fire‐bellied toad (*Bombina bombina*) is a highly threatened amphibian species, especially in Northern Europe. After the last glacial period, *B. bombina* recolonized the European continent out of putative refugia near the Black Sea in two distinct expansions (Fijarczyk et al., 2011; Hofman et al., 2007). Mountain ranges, in particular the Carpathian Mountains and Sudetes, presented natural barriers for *B. bombina* during the recolonization process, resulting in distinct northern and southern evolutionary lineages (Hofman et al., 2007). Despite the species’ widespread distribution, population numbers have declined dramatically during the last decades, mainly due to the loss, pollution, and eutrophication of suitable aquatic habitats (Drews et al., 2011; Fog, 1996; Günther & Schneeweiss, 1996). This population decline is particularly pronounced in the Baltic region, at the species’ north‐western range margin. In Sweden, the species was considered to have become extinct around the 1960s (Andrén et al., 1984). There are indications of unreported releases in the 1960s and 1970s (Drews et al., 2011). As a conservation measure, the species was reintroduced in Sweden in the 1980s (Andrén & Nilson, 1995). Drastic population declines have been also recorded throughout Denmark, Germany, and Latvia (Fog, 1996; Günther & Schneeweiss, 1996; Kuzmin, 1996). These range margin populations exhibit lower genetic diversity and a greater genetic differentiation than more central populations at nuclear microsatellites (Schröder et al., 2012), mitochondrial genomes, and transcriptome‐wide single nucleotide polymorphisms (SNPs; De Cahsan et al., 2021). This is thought of as a consequence of their smaller effective population size and greater geographical isolation (“abundant center model”; reviewed in Eckert et al., 2008).

Previous studies uncovered introgression of Austrian toads into Northern German and Southern Swedish *B. bombina* populations, presumably caused by unreported and illegal releases (De Cahsan et al., 2021; Drews et al., 2011; Schröder et al., 2012). The fitness consequences of these introgressions have been unknown so far, as they could have led to a reduced fitness (outbreeding depression) in future generations or—alternatively—have functioned as a genetic rescue by supplying new gene variants.

To investigate the fitness impact of this introgression event, we present a study on MHC and HSP gene variability, as well as an assessment of fitness‐related phenotypic traits of the European fire‐bellied toad from Northern Germany, Austria, and Southern Sweden. We sought to reveal the relative fitnesses of highly introgressed populations of *B. bombina* in comparison to putatively nonintrogressed populations as well as the number and the distribution of MHC II and HSP70 kDa alleles as candidates for putative local adaptation.

## 2 METHODS

### 2.1 Samples and DNA extraction

We extracted DNA from 284 samples from 17 different *B. bombina* populations from Southern Sweden (Skåne), Northern Germany (Schleswig‐Holstein), and Eastern Austria in 2016 (Tables A1 and A6, Figure A1 in Appendix S1). Genomic DNA was extracted from buccal swabs (Sweden and Austria) or toe clips (Germany) with the DNeasy™ blood and tissue extraction kit (QIAGEN, Hilden, Germany) following a modified version of the manufacturer's protocol (Table A2 in Appendix S1). To ensure an adequate final concentration of DNA, the amount of ATL‐, AL‐buffer and Proteinase K was doubled and the elution step was performed twice with 30 µl AE‐buffer.

### 2.2 Morphometric measurements

We measured body mass as well as the body length from snout to vent of each single toad in the field in 272 toads from 14 populations. To assess the individual body condition of the toads, we calculated the Scaled Mass Index (SMI) as described by Peig and Green (2009). This widely used method is based on a regression model and has been shown to be a suitable indicator for body condition, especially in amphibians (Jakob et al., 1996; Peig & Green, 2009, 2010). It provides a comparable measure across specimens of different sizes by rescaling body mass measures to a standard length. A better body condition is associated with a higher reproductive success in anurans; therefore, a higher SMI can be interpreted as an indicator for a better reproductive performance potential of the respective individual (MacCracken & Stebbings, 2012). The scaling exponent bSMA was calculated in R from ln‐transformed data using the slope of the best fit line of an Ordinary Least Square (OLS‐) Regression and dividing the result by the Pearson's correlation coefficient, as suggested by MacCracken and Stebbings (2012). For the individual SMI calculation, we specified a bSMA of 0.924 and scaled all individuals down to a standard body length of 45mm.

We compared body length, body mass, and the SMI among five groups: Northern (German and Swedish) populations, which we found to have been genetically introgressed by Austrian toads, German and Swedish populations putatively free of genetic introgression (according to our control region data and De Cahsan et al., 2021), and Austrian individuals.

We statistically evaluated the morphometric data by analyses of variance (ANOVA) and associated pairwise comparisons of mean values among groups.

### 2.3 mtDNA analysis to assess the introgression status of each population

We amplified a 210‐bp fragment of the mitochondrial control region for 297 samples (Table A3, Figure A1 in Appendix S1) to assess the genetic introgression of southern (Austrian) mtDNA haplotypes into the Northern (German and Swedish) populations of *B. bombina* (De Cahsan et al., 2021; Schröder et al., 2012). Polymerase chain reactions (PCRs) were performed according to previously published protocols (Dolgener et al., 2012; Schröder et al., 2012). We cleaned amplified products using an ExoAP cleaning procedure, which was then followed by a sequencing reaction and Sanger sequencing (ABI PRISM 3130xl Genetic Analyzer Sequencer). Control region sequences were aligned with ClustalW (Larkin et al., 2007; Thompson et al., 2002) as implemented in Geneious v9.1.6 (Kearse et al., 2012). Mitochondrial haplotypes were assigned, where possible, in accordance with published data (Schröder et al., 2012), and newly discovered haplotypes were uploaded to GenBank with the Accession numbers MW504725–MW504728.

### 2.4 Amplification, amplicon sequencing, and postprocessing of MHC II and HSP genes

Using previously published primers Bobom_MHCIIexon2‐F2 and Bobom_MHCIIexon2_R1 (Hauswaldt et al., 2007), we amplified a 156 bp fragment of the MHC class IIB exon 2 (excluding the primer sequences) for 60 toad samples from 17 populations. For the HSP70 kDA, we amplified multiple fragments from five exons in total for the same 60 individuals, using newly developed *B. bombina* primers designed from coding sequences of the Asian fire‐bellied toad (*B. orientalis*; Accession number: FJ387575.1) and two African clawed frog (*Xenopus* sp.) sequences provided from Xenbase (Gene ID: *X. laevis* XB‐GENE‐485630 v6.0 gene model; *X. tropicalis* XB‐GENE‐485626 v8.0 gene model) for the identification of the exon/intron margins (Table A4 in Appendix S1). PCRs were run with different conditions dependent on the fragment being amplified (Table A5 in Appendix S1). We pooled all MHC and HSP amplicons in approximately equal amounts determined through the visual inspection of band intensities on a 1.5% agarose gel. Individual amplicon pools were then built into 60 double‐indexed Illumina libraries following a previously published protocol (Meyer & Kircher, 2010), with some minor modifications (Fortes & Paijmans, 2015). We additionally included two library blanks. All libraries were sequenced in house on an Illumina MiSeq (Paired‐end 300 cycles) at the University of Potsdam, Germany. To balance out our low diversity libraries, we additionally ran a PHiX v2 control on the sequencing reaction.

We trimmed primer and Illumina adapter sequences from the resultant sequencing reads and removed reads shorter than 80 bp from all 60 samples using the software cutadapt v1.12 (Martin, 2011). Overlapping paired‐end reads were merged with a minimum overlap of 10 bp using FLASH v1.2.10 (Magoč & Salzberg, 2011). We further removed all bases with a quality score of less than 25 using prinseq (Schmieder & Edwards, 2011). Finally, we assessed the read quality with FastQC v4.1.2 (Andrews, 2010). Due to insufficient quality, two individuals were removed from further analysis of the HSP70 kDA (S84, A102). We repeated sequencing for several samples for the MHC II locus due to insufficient read depth and ended up with a representation of 59 toad individuals (for details Table A6 in Appendix S1). With the successfully merged reads, we then ran two independent mapping runs for MHC and HSP, respectively, specifying the burrow wheeler algorithm (BWA) v0.7.4‐r385 (Li & Durbin, 2009) and default parameters. One mapping was performed using previously published reference MHC II exon 2 sequences of *Bombina bombina* from NCBI (Accession numbers: EF210743.1, EF210742.1, EF210741.1, EF210740.1, EF210739.1, EF210738.1, EF210737.1, EF210736.1) and the other used a *B. bombina* HSP70 kDa reference sequence retrieved via Sanger sequencing during primer development. We then parsed the resultant mapping files using SAMtools v1.3.1 (Li et al., 2009).

### 2.5 MHC II exon 2

#### 2.5.1 Genotyping pipeline and validation of alleles

As often encountered in MHC analyses of nonmodel organisms, the number of MHC II loci in *B. bombina* is still unknown. The applied primers target several loci (Hauswaldt et al., 2007). To identify true alleles, we first counted for each individual the number of identical reads and removed all sequence types that made up less than 10% of the total number of reads per individual, as this fraction is likely to contain sequencing or PCR errors (Pfeiffer et al., 2018). We then aligned the retained reads with MAFFT (Katoh & Standley, 2013) specifying default parameters and trimmed all reads to the same length as our NCBI consensus reference sequence. From these trimmed reads, we inferred the number of alleles for each individual independently and checked for the co‐occurrence of alleles in multiple individuals. To minimize the possibility of false alleles occurring due to PCR or sequencing errors, remaining sequences were only kept if they were present in at least three different individuals and were therefore detected in three independent PCRs. As a final control step, we cross‐validated the inferred alleles with those inferred in Northern lineage Fire‐bellied toads via cloning and Sanger sequencing in a previous study (Drews et al., 2011; Pokorny, 2010). Our approach conceptually followed established protocols with regard to validation of alleles (Zagalska‐Neubauer et al., 2010) and the implementation of a cutoff of allele representation, below which a sequence was classified as artifact (Sommer et al., 2013). To further minimize the false discovery rate, we applied these established concepts more stringently: We demanded verification in 3 independent PCRs (as compared to 2 suggested by Zagalska‐Neubauer et al., 2010) and used a cutoff of 10% frequency below which a sequence was classified as artifact (as compared to the 5.4% of the most abundant artifact identified by Sommer et al., 2013). Our rationale was to build our inference on introgression resp. local adaptation upon robustly defined true MHC alleles (to the exclusion of artifacts erroneously taken as alleles). Our approach does not take into account rare alleles present only in single individuals and has hence an inherent tendency to underestimate allelic diversity.

#### 2.5.2 Circos Plot and allele frequency analyses

We counted the number of alleles found per individual and compared allele frequencies for each population. To highlight the presence and frequency of shared alleles, alleles shared between two countries were assigned individual colors, and co‐occurrences of alleles were visualized in a Circos plot (Krzywinski et al., 2009). Alleles present in all three locations were excluded from the Circos plot but included in a pie frequency charts to infer (putatively ancestral) ubiquitous alleles as well as (putatively derived) private alleles. For this analysis, MHC II exon 2 allele frequencies were calculated and pie charts were plotted into a map of each population's location.

#### 2.5.3 Translation and selection test

We translated the validated MHCII exon 2 alleles into their respective amino acid sequences and highlighted changes between sequences using Mega X v10.1.4 (Kumar et al., 2018; Stecher et al., 2020). We calculated the numbers of nonsynonymous substitutions per nonsynonymous site (dN) and synonymous substitutions per synonymous site (dS) to obtain the genewise dN/dS ratio using DnaSP v6 (Librado & Rozas, 2009) and tested it for significant deviation from 1 using a z‐test, as implemented in Mega. Under neutrality, dN and dS are expected to be equal, such that the dN/dS ratio is expected to be 1, while a ratio above or below 1 is indicative of positive or negative selection, respectively (e.g., Nielsen, 2005). We performed a dN/dS ratio test to detect positively selected codons, running the single‐likelihood ancestor counting (SLAC) and fixed effects likelihood (FEL) method as implemented in Datamonkey (http://classic.datamonkey.org) (Delport et al., 2010; Pond & Frost, 2005), an established approach to infer selection at MHC loci within species (e.g., Alcaide et al., 2014).

### 2.6 HSP70 kDa gene

#### 2.6.1 Genotyping pipeline and validation of alleles

After mapping, we performed a variant calling with SAMtools mpileup and bcftools v1.3.1 (Li, 2011; Li et al., 2009). We generated a consensus sequence on the basis of the called variants per individual and exon with a minimum coverage of 20 per called variant using vcfutils v1.3.1 (Li, 2011; Li et al., 2009). The consensus sequences were encoded with the standard IUPAC code for ambiguity for all heterozygous sites (Cornish‐Bowden, 1985).

We aligned the resultant 58 consensus sequences independently for each exon using ClustalW (Thompson et al., 2002) within Geneious v9.1.6 (Kearse et al., 2012). We excluded one individual (SH82) from further analyses, due to missing data in its exon sequences. This resulted in a total HSP exon sequence length of 1,174 bp for 57 samples.

#### 2.6.2 Allele network, Circos Plot, and allele frequencies

To identify variable positions and the heterozygosity level of the HSP70 kDa gene of *B. bombina*, we analyzed each exon independently using a sliding window approach implemented in DNAsp v6 (Librado & Rozas, 2009), specifying a step and window length of one. To reconstruct haploid genotypes from diploid DNA, we used the PHASE algorithm v2.1 (Stephens & Donnelly, 2003; Stephens et al., 2001) for all 57 samples. An allele network was computed from the resultant 114 haploid sequences spanning all five exons using the program popART v1.7 (Leigh & Bryant, 2015). We then assigned individuals into two groups, nonintrogressed or introgressed according to their source population. Subsequently, we performed a chi‐squared test of homogeneity to test for differences in observed allele numbers (Stanberry, 2013). In addition to the allele network and in order to assess the number of shared alleles between the three examined regions, a Circos plot was generated (Krzywinski et al., 2009). Allele frequencies were also included in a pie chart to infer (putatively ancestral) ubiquitous and (putatively derived) private alleles. HSP70 kDa allele frequencies were calculated and plotted as pie charts for each population independently.

#### 2.6.3 Translation and selection test

To identify putative amino acid and expressed protein variation of the HSP70 kDa gene, we translated the exons of *B. bombina* into amino acids, applying the known reading frame of the HSP70 kDa gene from *B. orientalis* (Accession number: FJ387575.1) using Geneious v9.1.6 (Kearse et al., 2012). Only exon 5 exhibited a nonsynonymous substitution. We calculated the genewise dN/dS ratio for this exon (148 bp) using DnaSP v6 (Librado & Rozas, 2009) and tested it for significant deviation from 1 using a *z*‐test, as implemented in Mega. Because of the low frequency of inferred nonsynonymous substitutions (1 out of 17 polymorphic sites in exon 5, none in the other exons), no site‐specific selection test was performed.

## 3 RESULTS

### 3.1 Morphometric measurements

**FIGURE 1:**
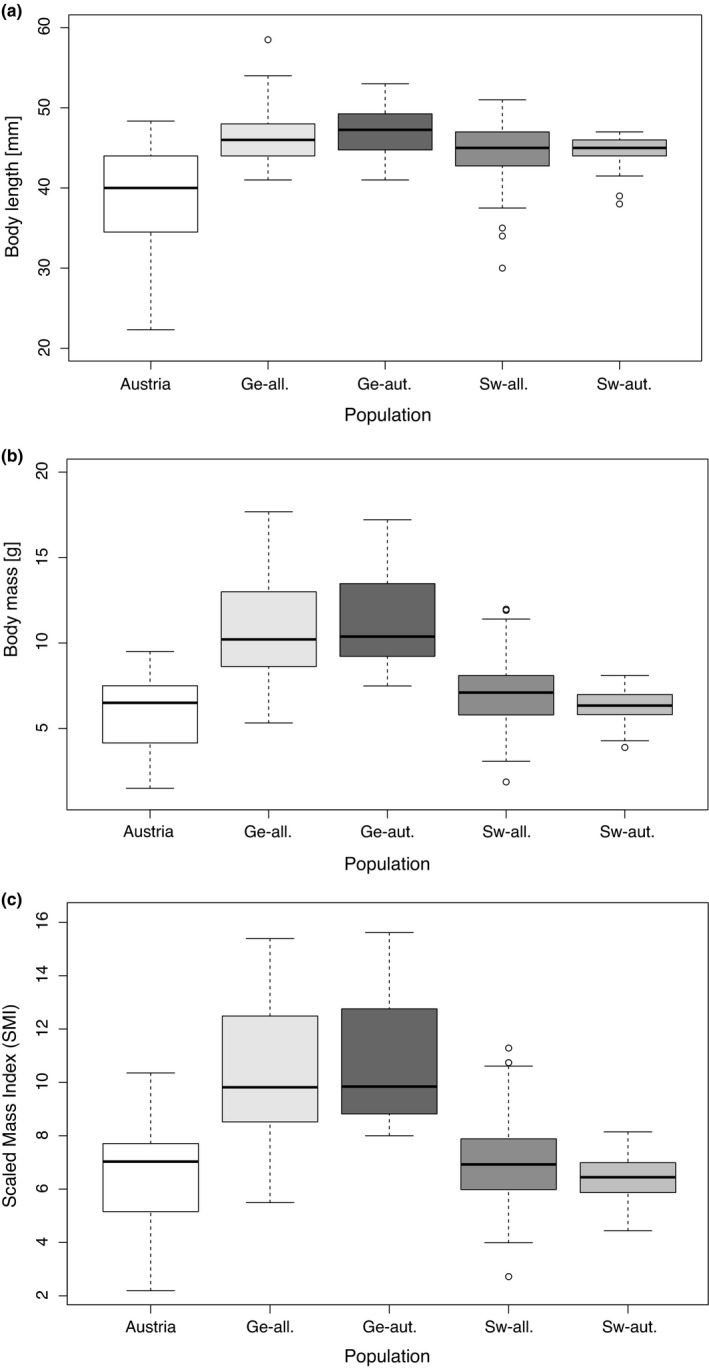
Boxplots of body length (a), body weight (b), and SMI (c) for 272 *B. bombina* individuals, comparison between Austrian populations (Austria), German introgressed (Ge‐all.), German nonintrogressed (Ge‐aut.), Swedish introgressed (Sw‐all.), and Swedish nonintrogressed (Sw‐aut.). The horizontal black bars represent median values, and whiskers refer to the minimum and maximum values observed within the data for each population. Potential outliers are shown as outlined circles

Southern lineage toads (Austria) were—on average—significantly smaller than Northern lineage toads (Figure 1a, Table A7 in Appendix S1). There were no differences in body length among Northern populations, neither between Germany and Sweden nor between introgressed and autochthonous. Austrian and autochthonous Swedish toads had a similar average body mass (5.7 g ± 0.2 g vs. 6.4 g ± 0.2 g [mean ± *SE*], respectively; Figure 1b,c; Table A8 in Appendix S1), while Swedish introgressed populations were significantly heavier than Austrian populations (7.2 g ± 0.3 g [mean ± *SE*], *p* = .002). In comparison, the German populations were significantly heavier and had a higher SMI than both the Austrian and the Swedish populations. There was no significant difference in body mass or SMI among introgressed and nonintrogressed populations in Germany.

### 3.2 mtDNA control region analysis to assess introgression status

Among the analyzed 297 specimens from 17 populations, we found 3 northern lineage (AA*) and 4 southern lineage (DEG*) haplotypes (Figure A1 in Appendix S1; cf. Schröder et al., 2012 for lineage definition). This analysis confirmed southern lineage introgression for 6 populations (3 each of Germany and Sweden) and indicated lack of introgression for 3 populations (2 German; 1 Swedish) (Table A6, and Figure A1 in Appendix S1). The German population of Eutin was nonetheless considered introgressed, as introgression had been previously verified (De Cahsan et al., 2021), rendering only two populations (on the island of Fehmarn in Germany and at Gislöv in Southern Sweden) without any sign of introgression (hereafter called nonintrogressed/autochthonous).

### 3.3 MHC II

We found 19 unique alleles coding for 19 unique protein sequences (i.e., no alleles differing by synonymous mutations only; Figure A2a in Appendix S1). The genewise dN/dS ratio was 0.248, indicative of negative (purifying) selection (*p* = .03, *z*‐test). In the site‐specific analysis, however, the single‐likelihood ancestor counting (SLAC) method identified two sites under positive/diversifying selection and one under negative/purifying selection. The fixed effects likelihood (FEL) method identified the same three sites under selection plus additional three sites under positive, and additional two sites under negative selection. Almost all sites under positive selection, except for one, are located in putative antigen‐binding sites (ABS) known from human leukocyte antigen genes (HLA), which are part of the MHC gene complex (Figure A2b in Appendix S1). All sites under negative selection were found outside the ABS of HLA.

**FIGURE 2:**
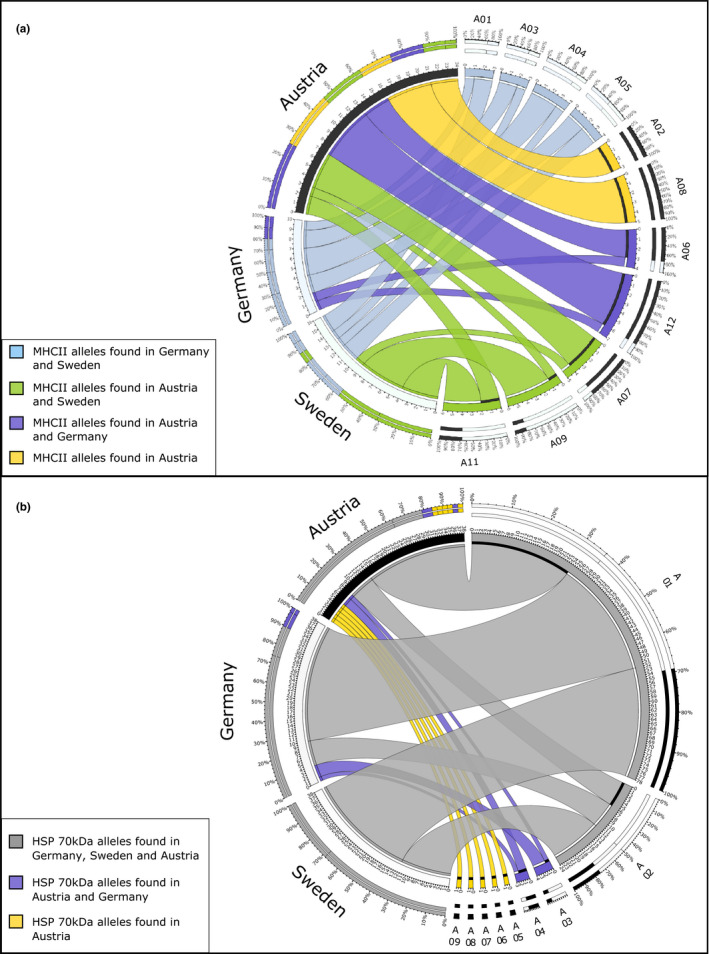
Circos plots of allele distribution among sampling regions for (a) MHC II exon 2. Note that ubiquitous alleles shared among all three regions are not included. (b) HSP70 kDa

To investigate the abundance of shared exon 2 alleles among regions (i.e., Austria, Northern Germany, and Southern Sweden), we plotted the proportion of shared alleles within and between regions using a Circos plot (Figure 2a). In Austria, 32% of all individuals carried private Austrian alleles. In comparison, the majority of individuals in German populations (80%) carried private northern alleles (alleles only found in northern lineage populations). The remaining 20% of alleles in Germany are shared alleles, present also in Austrian individuals. In Sweden, we found northern private alleles at a total frequency of approximately 40%. The other 60% are alleles shared among Sweden and Austria. Austria is the region with the highest MHC II exon 2 allele diversity. We did not find private Swedish or private German MHC II exon 2 alleles in our dataset.

We counted the total number of MHC II exon 2 alleles in Austrian, Swedish, German, and combined northern (German and Swedish) populations for each individual and per group (Table 1). The average number of MHC II exon 2 alleles per individual for all Austrian populations is 3, which is the lowest average allele count found in all of the investigated samples from the three countries. We found the highest average allele number (5.27) in the introgressed Swedish populations. Introgressed populations show higher average allele counts in both Sweden and Germany than their nonintrogressed counterparts. The Mann–Whitney U test identified a significant difference in average allele counts between Swedish introgressed and nonintrogressed populations, Austrian and Swedish introgressed, Austrian and northern introgressed, and northern introgressed and nonintrogressed populations (Table 2). The highest allele number found in a single individual (9) was detected in Mölle, Sweden. The lowest allele number (1) was found in two individuals, one from Weiden, Austria, and one from Fehmarn, Germany. We did not find a correlation between allele counts per individual and body condition (SMI; Figures A3 and A4 in Appendix S1).

**TABLE 1:** Allele numbers at MHC II exon 2 per individual (averaged for groups/populations)

Group/population	Average allele number (allele count), min: 1 – max: 9
Austria (Au)	3.00
Introgressed Sweden (Sw‐all.)	5.27
Nonintrogressed Sweden (Sw‐aut.)	3.14
Introgressed Germany (Ge‐all.)	4.09
Nonintrogressed Germany (Ge‐aut.)	3.71
Introgressed (NORTH‐all.)	4.68
Nonintrogressed (NORTH‐aut.)	3.43

**TABLE 2:** Mann–Whitney U test for significance of differences between average allele numbers at MHC II exon 2 per individual for each pairwise comparison

Comparison	U	Z‐score	*p*‐value
Austria versus Sweden (nonintrogressed)	35.5	−0.226	.818
Austria versus Sweden (introgressed)	21	−2.561	.011*
Austria versus Germany (nonintrogressed)	29.5	−0.770	.441
Austria versus Germany (introgressed)	33	−1.773	.077
Austria versus North (nonintrogressed)	65	0.630	.530
Austria versus North (introgressed)	54	2.540	.011*
Sweden (nonintrogressed) versus Sweden (introgressed)	12	2.355	.019*
Germany (nonintrogressed) versus Germany (introgressed)	33.5	0.408	.682
North (nonintrogressed) versus North (introgressed)	85.5	2.206	.027*

* Depicts significance at *p* < .05.

**FIGURE 3:**
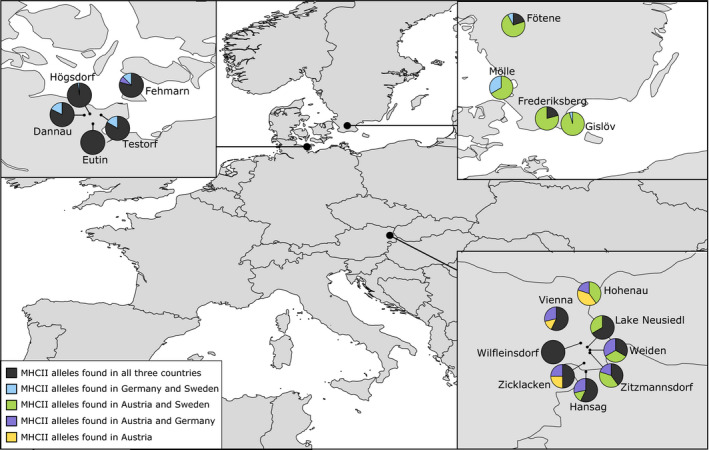
MHC II exon 2 relative allele frequencies for each sampling site (population) for 58 *Bombina bombina* individuals, grouped and colored according to their occurrence in one or more regions

We found ubiquitous alleles (i.e., alleles present in all three study regions Austria, Germany, Sweden) at high frequency in all German and the majority of the Austrian populations. In contrast, in Sweden, these ubiquitous alleles were only present in two out of four populations, and at lower frequencies (Figure 3). In Sweden, specific northern alleles (light blue in Figure 3) occurred at low frequency or were even absent (Frederiksberg), and the majority of alleles were shared only with Austria, but not with Northern Germany. We found private Austrian MHC II exon 2 alleles in three out of eight Austrian *B. bombina* populations, all located northwest of lake Neusiedl and in Vienna.

### 3.4 HSP70 kDa

**FIGURE 4:**
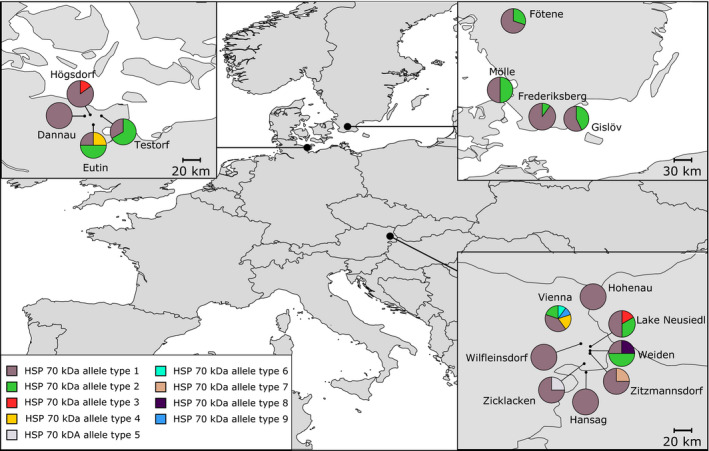
Allele frequencies of the HSP70 kDa gene for all 57 *Bombina bombina* individuals for all sampling locations

The different exon fragments of the HSP70 kDa gene differed substantially in their number of polymorphic sites. We found one polymorphic site in exon 1, none in exons 2 and 3, seven in exon 4, and 17 in exon 5. In order to detect differences between alleles from nonintrogressed and introgressed populations, an allele network including all the 25 polymorphic sites was constructed. Nine different alleles were identified (Figure A5 in Appendix S1). We confirmed significant differences between the allele frequencies of the different populations with the chi‐squared test of homogeneity (Table 3). The allele network shows a star‐like pattern, with the most abundant allele in the center, found 78 times. Allele types 5–9 were only detected in Austrian individuals, and these alleles were highly differentiated from the other allele types found in the northern populations. The allele network did not reveal a clear geographic pattern regarding sampling regions, nor did it mirror the classification into nonintrogressed and introgressed. However, we were able to identify some alleles that are only shared among Austrian and German introgressed populations (Allele type 3 and 4, Figure A6 in Appendix S1). We found the most abundant alleles (Allele 1 and 2) to be shared among all three examined regions (Sweden, Austria, Northern Germany) (Figure A6 in Appendix S1 and Figure 2b). However, in the southern lineage and the northern lineage introgressed populations, allele 1 is 3–5 times more abundant than allele 2, whereas in the nonintrogressed population, both occur at about equal frequencies (Table 3). Sweden has the lowest allele diversity with only two different alleles present, whereas Germany shows a more diverse allelic pattern (Figure 2b). We detected the highest allele diversity in Austrian populations, with a total of five private alleles (11% of total allele diversity) (Figure 4). Within the Austrian populations, the Vienna population is particularly diverse with three private alleles present (Allele 4, 8, 9; Figure 4).

**TABLE 3:** HSP70 kDa allele frequencies in different sets of populations. The allele frequencies are significantly different among the sets (Chi‐squared test of homogeneity)

Population	Allele type									
	2n	1	2	3	4	5	6	7	8	9
Austria nonintrogressed	38	24	6	1	2	1	1	1	1	1
German introgressed	38	28	6	3	1	0	0	0	0	0
Sweden nonintrogressed	14	8	6	0	0	0	0	0	0	0
Sweden introgressed	24	18	6	0	0	0	0	0	0	0

Note

*p* = .0272 (Chi‐squared test of homogeneity).

Using the known reading frame of the Asian fire‐bellied toad (Accession number: FJ387575.1, NCBI), we translated each *B. bombina* exon into a protein sequence to look for expressed variation. The transformation into protein structures identified two alleles (allele type 8 and 9) carrying the same expressed polymorphism (position 45 in exon 5, replacing leucine (L) by phenylalanine (F) within a leucine‐rich region (LRR)). Each of these alleles occurred only in one out of the 57 analyzed individuals. The genewise dN/dS ratio was 0.038. As we only found a single nonsynonymous substitution, this estimate is not precise. Still, it deviates highly significantly from the neutral expectation (*p* < .001, *z*‐test) and is hence indicative of negative (purifying) selection on HSP70 kDA in the geographical regions analyzed here.

## 4 DISCUSSION

We analyzed body condition (as a proxy for fitness) and genetic variation at expressed genes in *B. bombina* originating from two northern countries, both independently introgressed by toads from the southern lineage (introgression in Schleswig‐Holstein shortly before 2001; introgression in Sweden likely in the 1960s to 1970s; Drews et al., 2011). When comparing the Austrian source populations to Swedish populations, introgressed populations had a significantly higher weight, a pattern not found in the nonintrogressed populations. German populations (both introgressed and nonintrogressed) had an increased body condition (calculated using the SMI), relative to both Austrian and Swedish ones. Body condition is linked to the reproductive output potential in anurans (Johansson et al., 2007; Luquet, David, et al., 2011; Luquet, Léna, et al., 2011; Luquet et al., 2013) and is strongly influenced by the environment (Bonnet et al., 2001; Douhard et al., 2013; Ozgul et al., 2010; Toïgo et al., 2006). A larger body size in anuran males, for example, can increase mating success, as it helps to secure the best nest sites and correlates with low embryonic mortality (Howard, 1978). The same study suggests that anuran females with a larger body length can reproduce more often per year. A study on Japanese amphibian females revealed a link between female standard weight and clutch weight (Kuramoto, 1978), while other studies found a correlation between the female body length and clutch size (Berven, 1982; Pettus & Angleton, 1967; Woolbright, 1983). A better body condition may also lead to choosier and riskier behavior in females, as it enables them to swim longer distances to reach a particularly attractive, calling male in the presence of predators (Kuczynski et al., 2017).

Our study reveals putatively fitness‐related morphometric traits to be dependent on geographic origin, likely reflecting different environmental conditions. Among the northern lineages, the northernmost (Swedish) population score significantly lower in average morphometric trait values (weight, condition index) when compared to their Northern German counterparts. These Swedish populations mark the northern margin of the species distribution and may hence struggle with suboptimal habitats and adverse climatic conditions. Our finding supports the hypothesis that introgression of southern genotypes into these northernmost *B. bombina* populations may lead to an increased fitness (here, increased body weight). The introgression may have provided adaptive alleles at many loci (De Cahsan et al., 2021) which were subsequently favored by natural selection. Interestingly, while fire‐bellied toad populations introgressed by conspecifics of another evolutionary lineage (here, the southern lineage into the northern lineage) may gain a fitness benefit, species hybrids between the fire‐bellied toad (*B. bombina*) and the yellow‐bellied toad (*B. variegata*)—which occur where the species’ distribution ranges overlap—appear to be selected against (Kruuk et al., 1999).

To further evaluate potential adaptive effects of introgression, we assessed the genetic diversity of genes potentially involved in local adaptation (MHC II, HSP). We find northern *B. bombina* populations to be generally genetically less diverse at these loci, when compared to conspecific southern populations in Austria. These lower levels of genetic diversity could reflect a combination of local adaptation, colonization history, edge effects, and declines in population size during the last century. A reduced genetic variability of these northern populations has also been observed at neutral molecular markers (Hofman et al., 2007; Pabijan et al., 2013; Schröder et al., 2012). Furthermore, as these regions represent the species’ north‐western range margin, fast‐paced colonization processes followed by repeated founder effects and inbreeding might have decreased the genetic diversity and caused genetic erosion, that is, loss of adaptive genetic material in these populations (Hewitt, 2000; Ibrahim et al., 1996).

Introgression from a geographically distant lineage into a genetically isolated population instantly increases the diversity in a population's gene pool (e.g., Hedrick, 1995; Madsen et al., 1999; Mansfield & Land, 2002). This could either promote adaptation to changes in environmental/climatic conditions (Arnold & Kunte, 2017; Janes & Hamilton, 2017) or disrupt locally adapted gene assemblages by an influx of foreign alleles (outbreeding depression) (Edmands, 2007).

Surprisingly, few MHC II exon 2 alleles were shared among German and Swedish *B. bombina* populations. In particular, Swedish populations were dominated by alleles never found in Northern Germany, but shared with the Austrian population. This could reflect a different introgression history. In Sweden, where a translocation of Austrian toads happened at least 30 years earlier, the same shared MHC II exon 2 alleles with the Austrian population are present in all of the four populations, including the Gislöv/Glimming population where no signs of a mitochondrial introgression from Austria has been found (Figure A1 in Appendix S1). Therefore, these may also represent ancestral alleles. Interestingly, introgressed Swedish populations had significantly higher per individual MHC allele numbers than Austria. This might be another indication for introgression of alleles from the south increasing the numbers of already present alleles in Sweden.

To identify relevant substitutions under selective pressure that may differ among populations, we performed selection tests. Purifying selection was inferred for the MHC II exon 2 in the genewise analysis. However, through a comparison of the ratio of nonsynonymous and synonymous substitutions, we detected multiple sites under positive selection in the MHC II. This result was not unexpected, as in MHC class II molecules, the exon 2 encodes parts of the antigen‐binding sites (ABS) and an excess of nonsynonymous substitutions is commonly observed, as typical for protein domains with antigen‐binding function (Bernatchez & Landry, 2003; Brown et al., 1993; Hughes & Nei, 1988; Piertney & Oliver, 2006).

In contrast to the MHC results, we find a low number of polymorphic sites within the HSP70 kDa gene. This result was in line with evolutionary theory, which generally predicts a higher substitution rate for synonymous substitutions than nonsynonymous ones in coding regions, as the latter are typically subject to negative (purifying) selection (Hughes et al., 2001), as also confirmed in our study by the low number of nonsynonymous versus synonymous substitutions (dN/dS significantly lower than 1). Despite the relatively low number of polymorphic sites, we found a single unique nonsynonymous substitution within exon 5 of the HSP70 kDa gene only present in Austria, conferring an amino acid alteration from leucine to phenylalanine. This expressed substitution within exon 5 is located within a leucine‐rich region (LLR) known to play an important role in chaperone protein‐protein interactions (Kobe & Deisenhofer, 1994). HSPs undertaking chaperone functions are known to assist in or correct the folding of damaged proteins, as well as stabilizing newly synthesized ones (De Maio, 1999). This is often the case when organisms are experiencing stressful conditions, such as exposure to unusual temperatures or UV light (Ritossa, 1962). However, whether the inferred expressed polymorphism has any functional relevance in this regard remains to be evaluated.

Generally, HSP genetic variability was considerably higher in the southern lineage than in the northern lineage populations and two ubiquitous alleles (allele 1 and 2) consistently occurred at high frequencies across all populations. The ratio among these alleles was shifted toward allele 1 in Austria and the northern introgressed populations. This and the occurrence of a specific HSP allele (type 3, cf. Figure 4) in the German population of Högsdorf (a population with high levels of introgression from the southern lineage; De Cahsan et al., 2021; Schröder et al., 2012) may indicate introgression effects also at this locus. The introgression has not introduced gene variants with expressed differences. However, at HSP70, local adaptation in amphibians may rather regard expression levels than expressed differences in the mature protein (Sørensen et al., 2009). Such differences could be caused by heritable differences in both *cis* and *trans* gene regulatory elements. It would be hence valuable to analyze the 5’ promoter region as well as the expression levels of the different HSP70 alleles across autochthonous and introgressed populations in future studies.

Our study on wild populations of *B. bombina* shows the complex genetic consequences of translocating specimens, which should be considered in conservation schemes. Local gene assemblages can potentially be disrupted by the influx of foreign alleles, threatening locally adapted populations. However, as highly fragmented and isolated populations at a species range margin often lack genetic diversity, beneficial effects of translocation may prevail, as new gene variants from distant populations could promote adaptive genetic exchange (Arnold & Kunte, 2017). This may have been the case in our study, as we found some indication of increased body condition of introgressed Swedish fire‐bellied toad populations compared to nonintrogressed populations. This study also shows that the MHC II locus is a good candidate gene to investigate local adaptation, as the Austrian gene introgression into northern fire‐bellied toad populations clearly elevated allelic diversity at this locus where variation is indeed considered adaptive (Sommer, 2005). The conserved HSP70 kDA gene did not show much variation. Here, local adaptation may be rather found as expression differences in heat shock protein genes among populations under different climatic conditions. Indeed, expression differences of the HSP70 kDa may play a role during developmental processes as has been shown for other amphibians (Angelier et al., 1996; Billoud et al., 1993; Heikkila, 2010; Sørensen et al., 2009). However, as the physiological responses and underlying gene networks to cope with defined environmental parameters (here, local pathogens/parasites and temperature) are not well studied in nonmodel organisms, it is important to evaluate the effect of introgression at additional candidate genes recovered from genome‐ or transcriptome‐wide studies. More loci will enable a more accurate assessment of local adaptation and the potential impact of introgression, regardless of whether the translocation constitutes a planned conservation measure or an unreported release (as in our case). With genome/transcriptome data at hand, it is possible to disentangle gene loci where introgressed alleles prevail and where autochthonous alleles are retained to better understand the dynamics of introgressive hybridization and its relevance for conservation.

## CONFLICT OF INTEREST

The authors declare no conflict of interest.

## AUTHOR CONTRIBUTIONS

**Binia De Cahsan:** Conceptualization (supporting); Data curation (lead); Formal analysis (equal); Investigation (equal); Visualization (equal); Writing‐original draft (lead); Writing‐review & editing (equal). **Katrin Kiemel:** Data curation (supporting); Formal analysis (equal); Investigation (equal); Visualization (equal); Writing‐original draft (supporting); Writing‐review & editing (equal). **Michael Westbury:** Formal analysis (supporting); Investigation (supporting); Supervision (supporting); Writing‐original draft (supporting); Writing‐review & editing (equal). **Maike Lauritsen:** Data curation (supporting); Formal analysis (supporting); Investigation (supporting); Visualization (supporting); Writing‐review & editing (supporting). **Marijke Autenrieth:** Formal analysis (supporting); Visualization (supporting); Writing‐review & editing (supporting). **Günter Gollmann:** Resources (supporting); Writing‐review & editing (supporting). **Silke Schweiger:** Resources (supporting); Writing‐review & editing (supporting). **Marika Stenberg:** Resources (supporting); Writing‐review & editing (supporting). **Per Nyström:** Resources (supporting); Writing‐review & editing (supporting). **Hauke Drews:** Resources (supporting); Writing‐review & editing (supporting). **Ralph Tiedemann:** Conceptualization (lead); Data curation (supporting); Formal analysis (supporting); Funding acquisition (lead); Investigation (supporting); Project administration (lead); Resources (lead); Supervision (lead); Writing‐original draft (supporting); Writing‐review & editing (equal).

## Supporting information

Appendix S1

## Data Availability

DNA sequences: GenBank accessions mtDNA/control region: MW504722–MW504722; MHC II: MW491359–MW491377; HSP 70 kDa: MW525223–MW525254. Sampling locations and morphological data: Table A1 and A6 in Appendix S1.
