# Supplementary material for: Southern introgression increases adaptive immune gene variability in northern range margin populations of Fire‐bellied toad
Source: Ecol Evol. 2021 Jun 27;11(14):9776–90. doi: 10.1002/ece3.7805 (PMC8293767; doi:10.1002/ece3.7805)
Supplement: Supplementary file 1 — Appendix S1 [file ECE3-11-9776-s001.docx]

**Appendix**

**Tables**

**Table A1:** Overview of sampling locations, their respective GPS coordinates, introgression status and sample sizes used for the calculation of the SMI and each individual genetic marker in this study.

| **Coordinates** | **Country** | **Introgression**  **status** | **Site** | **SMI** | **Control region** | **MHC** | **HSP** |
| --- | --- | --- | --- | --- | --- | --- | --- |
| 55.52, 13.80 | Southern Sweden | introgressed | Fredriksberg/Skogshuset | 21 | 17 | 5 | 5 |
| 55.49, 14.27 |  | non-introgressed | Gislov/Glimminge | 34 | 24 | 7 | 7 |
| 56.27, 12.50 |  | introgressed | Moelle | 21 | 18 | 1 | 2 |
| 58.07, 12.79 |  | introgressed | Foetene | 13 | 11 | 5 | 5 |
| 54.23, 10.53 | Northern Germany | introgressed | Dannau | 15 | 19 | 4 | 4 |
| 54.51, 11.12 |  | non-introgressed | Fehmarn | 20 | 11 | 7 | 0 |
| 54.24, 10.60 |  | introgressed | Hoegsdorf | 22 | 11 | 10 | 10 |
| 54.12, 10.64 |  | introgressed | Eutin | 10 | 4 | 2 | 2 |
| 54.23, 10.74 |  | introgressed | Testdorf | 26 | 12 | 2 | 3 |
| 48.18, 16.49 | Austria |  | *Wien-1* | *10* | *7* | *1* | *1* |
| 48.18, 16.49 |  |  | *Wien-2* | *12* | *12* | *1* | *1* |
| 48.18, 16.49 |  |  | *Wien-3* | *14* | *12* | *0* | *0* |
| 48.18, 16.49 |  |  | *Wien-4* | *7* | *7* | *1* | *1* |
| 48.18, 16.49 |  |  | *Wien-5* | *7* | *7* | *0* | *1* |
| 48.18, 16.49 |  |  | Wien-1-5 | 50 | 45 | 3 | 4 |
| 48.60, 16.91 |  |  | Hohenau an der March | 0 | 8 | 1 | 1 |
| 47.76, 16.78 |  |  | Zickladen | 10 | 9 | 1 | 2 |
| 47.92, 16.85 |  |  | Weiden | 11 | 9 | 2 | 2 |
| 47.89, 16.85 |  |  | Zitzmanndorfer Wiesen | 9 | 9 | 2 | 2 |
| 47.66, 16.81 |  |  | Hansag | 10 | 9 | 2 | 2 |
| 48.00, 16.74 |  |  | Wilfleinsdorf | 0 | 8 | 1 | 1 |
| 47.83, 16.76 |  |  | Neusiedler See | 0 | 0 | 2 | 3 |

**Table A2:** Modification of the QIAGEN standard protocol of the DNeasy™ Tissue Kit for animal tissue.

| **Step** | **Standard protocol** | **Changes** |
| --- | --- | --- |
| **1**  **2**  **3**  **7** | 180 µl ATL-buffer  20 µl Proteinase K  200 µl AL-buffer  100 µl AE-buffer | 360 µl ATL-buffer  40 µl Proteinase K  400 µl AL-buffer  2x 30 µl AE-buffer |
|  |  |  |

**Table A3:** Haplotype frequencies of the mitochondrial control region for all individuals from Germany, Austria and Sweden.

|  | **Germany** | | | | | **Austria** | | | | | | | **Sweden** | | | |
| --- | --- | --- | --- | --- | --- | --- | --- | --- | --- | --- | --- | --- | --- | --- | --- | --- |
| **Haplotype** | **Dannau** | **Eutin** | **Fehmarn** | **Högsdorf** | **Testorf** | **Wien** | **Hohenau** | **Zicklacken** | **Weiden** | **Zitzmannsdorf** | **Hansag** | **Wilfleinsdorf** | **Frederiksberg** | **Mölle** | **Fötene** | **Gislöv** |
| **DEG** | 7 | 0 | 0 | 22 | 6 | 22 | 7 | 7 | 8 | 10 | 8 | 8 | 12 | 17 | 8 | 0 |
| **AAC** | 22 | 0 | 3 | 3 | 4 | 0 | 0 | 0 | 0 | 0 | 0 | 0 | 4 | 1 | 2 | 22 |
| **AAB** | 4 | 21 | 23 | 1 | 20 | 0 | 0 | 0 | 0 | 0 | 0 | 0 | 0 | 0 | 0 | 0 |
| **DEG-T** | 0 | 0 | 0 | 0 | 0 | 0 | 1 | 2 | 0 | 0 | 2 | 0 | 0 | 0 | 0 | 0 |
| **DEG-A** | 0 | 0 | 0 | 0 | 0 | 4 | 0 | 0 | 0 | 0 | 0 | 0 | 0 | 0 | 0 | 0 |
| **AAC-A** | 0 | 0 | 0 | 0 | 0 | 0 | 0 | 0 | 0 | 0 | 0 | 0 | 0 | 0 | 1 | 2 |
| ***N*** | **33** | **21** | **26** | **26** | **30** | **26** | **8** | **9** | **8** | **10** | **10** | **8** | **16** | **18** | **11** | **24** |

**Table A4:** Primer pairs for all exons and exon fragments of the HSP70 kDa gene developed in this study.

| **Name** | **Length [bp]** | **Primer sequence 5’ – 3’** | **Product size [bp]** |
| --- | --- | --- | --- |
| B.ori_Ex1 F | 21 | GTGTCTTCCAGCATGGCAAAG | 150 |
| B.ori_Ex1 R | 24 | CAAACACGGTATTCTGAGGGTTAA |  |
| B.ori_Ex2 F | 24 | ATTGGCAGAAAATATGACGAACCC | 134 |
| B.ori_Ex2 R | 20 | TCTTCAGGAGAGAAGCACTT |  |
| B.ori_Ex3 F | 25 | CTTTAAATCAGCCTGTAACCAATGC | 169 |
| B.ori_Ex3 R | 26 | GTGGAACTCTTATCAAGTCCATAAGC |  |
| B.b_Ex4.1 F | 27 | ACATAATGTTCTGATTTTTGACCTGGG | 151 |
| B.b_Ex4.1 R | 24 | TACAAAGTGGTTCACCATACGGTT |  |
| B.b_Ex4.2v.2 F | 21 | CTTTGAGGTTAAAGCCACAGC | 225 |
| B.b_Ex4.2v.2 R | 22 | GCCCTGGTGATAGAGGTGTAAA |  |
| B.b_Ex4.3 F | 22 | TTGAGAACAGCCTGTGAGAGAG | 214 |
| B.b_Ex4.3 R | 22 | TATCGTGGATCTGTGCCTTGTC |  |
| B.b_Ex4.4 F | 22 | GGCTCGCTTTGAAGAACTATGC | 215 |
| B.b_Ex4.4 R | 22 | GGCTACAGCTTCATCTGGATTG |  |
| B.b_Ex5 F | 24 | CAATTCTGATGGGTGACAAGTCTG | 150 |
| B.b_Ex5 R | 24 | ATCTGAGTTTGCTTGGTTGGAATG |  |
|  |  |  |  |

**Table A5:** Polymerase Chain reaction (PCR) conditions for the HSP70 kDa of *B. bombina* for each exon/exon fragment.

| **Reaction step** | **Temperature [°C]** | | **Time [min]** |
| --- | --- | --- | --- |
| Initial denaturation | 95.0 | | 1 |
| Denaturation | 95.0 | | 0.25 |
| Primer annealing | B.ori_Ex1 | 54.8 | \| 1 \| \| --- \|  \| 35x \| \| --- \| |
|  | B.ori_Ex2 | 52.7 |  |
|  | B.ori_Ex3 | 50.1 |  |
|  | B.b_Ex4.1 | 53.9 |  |
|  | B.b_Ex4.2v.2 |  |  |
|  | B.b_Ex4.3 |  |  |
|  | B.b_Ex4.4 |  |  |
|  | B.b_Ex5 | 55.6 |  |
| Elongation | 72.0 | | 1 |
| Final Elongation | 72.0 | | 0.1 |
| Pause | 10.0 | | ∞ |

**Table A6:** Overview of all samples, their respective population and country of origin, morphological data and genetic markers analysed.

|  |  |  |  | **Morphological data** | | | **Genetic markers (haplotypes and alleles)** | | |
| --- | --- | --- | --- | --- | --- | --- | --- | --- | --- |
| **Sample ID** | **Population** | **Country of origin** | **autochthonous/allochthonus** | **Body weight [g]** | **Body length [mm]** | **SMI** | **mtDNA/Controlregion HT** | **nucDNA/**  **MHCII** | **nucDNA/HSP70 kDa** |
| SW-G01 | SW-Frederiksberg/Skogshuset | Sweden | allochthonous | 6,76 | 44 | 6,902 | DEG |  |  |
| SW-G02 | SW-Frederiksberg/Skogshuset | Sweden | allochthonous | 5,86 | 44,4 | 5,933 | AAC |  |  |
| SW-G03 | SW-Frederiksberg/Skogshuset | Sweden | allochthonous | 5,72 | 42,5 | 6,030 | DEG |  |  |
| SW-G04 | SW-Frederiksberg/Skogshuset | Sweden | allochthonous | 7,29 | 43 | 7,603 | AAC |  |  |
| SW-G05 | SW-Frederiksberg/Skogshuset | Sweden | allochthonous | 3,58 | 35 | 4,515 | DEG | Allele 09,11,15,17,18 | 1/1 |
| SW-G06 | SW-Frederiksberg/Skogshuset | Sweden | allochthonous | 7,02 | 47 | 6,744 | DEG | Allele 15,17,18,19 | 1/1 |
| SW-G07 | SW-Frederiksberg/Skogshuset | Sweden | allochthonous | 10,03 | 51 | 8,935 | DEG | Allele 09,11,17,18,19 | 1/1 |
| SW-G08 | SW-Frederiksberg/Skogshuset | Sweden | allochthonous | 5,46 | 41,5 | 5,884 | DEG |  |  |
| SW-G09 | SW-Frederiksberg/Skogshuset | Sweden | allochthonous | 4,36 | 40 | 4,861 |  |  |  |
| SW-G10 | SW-Frederiksberg/Skogshuset | Sweden | allochthonous | 6,2 | 44,5 | 6,264 |  |  |  |
| SW-G11 | SW-Frederiksberg/Skogshuset | Sweden | allochthonous | 7,91 | 44 | 8,076 |  |  |  |
| SW-G12 | SW-Frederiksberg/Skogshuset | Sweden | allochthonous | 7,19 | 42 | 7,663 | DEG |  |  |
| SW-G13 | SW-Frederiksberg/Skogshuset | Sweden | allochthonous | 5,2 | 43 | 5,423 | DEG | Allele 15,18,19 | 1/1 |
| SW-G14 | SW-Frederiksberg/Skogshuset | Sweden | allochthonous | 7,08 | 43,5 | 7,305 | DEG |  |  |
| SW-G15 | SW-Frederiksberg/Skogshuset | Sweden | allochthonous | 5,89 | 43 | 6,143 | DEG |  |  |
| SW-G16 | SW-Frederiksberg/Skogshuset | Sweden | allochthonous | 4,17 | 38 | 4,875 | AAC | Allele 14,15,16,17,18,19 | 1/1 |
| SW-G17 | SW-Frederiksberg/Skogshuset | Sweden | allochthonous | 6,36 | 40 | 7,091 | DEG |  |  |
| SW-G18 | SW-Frederiksberg/Skogshuset | Sweden | allochthonous | 4,06 | 38 | 4,746 |  |  |  |
| SW-G19 | SW-Frederiksberg/Skogshuset | Sweden | allochthonous | 4,27 | 37,5 | 5,053 | DEG |  |  |
| SW-G20 | SW-Frederiksberg/Skogshuset | Sweden | allochthonous | 7,49 | 46 | 7,339 | AAC |  |  |
| SW-G21 | SW-Frederiksberg/Skogshuset | Sweden | allochthonous | 4,65 | 40 | 5,184 | DEG |  |  |
| SW-G22 | SW-Gisloev/Glimminge | Sweden | autochthonous | 6,15 | 45,5 | 6,088 | AAC |  |  |
| SW-G23 | SW-Gisloev/Glimminge | Sweden | autochthonous | 5,42 | 45,5 | 5,365 | AAC | Allele 15,18,19 | 1/2 |
| SW-G24 | SW-Gisloev/Glimminge | Sweden | autochthonous | 5,57 | 46,5 | 5,404 | AAC? | Allele 17 | 1/2 |
| SW-G25 | SW-Gisloev/Glimminge | Sweden | autochthonous | 6,26 | 43 | 6,528 |  |  |  |
| SW-G26 | SW-Gisloev/Glimminge | Sweden | autochthonous | 7,95 | 46 | 7,790 |  |  |  |
| SW-G27 | SW-Gisloev/Glimminge | Sweden | autochthonous | 6,31 | 44 | 6,442 |  |  |  |
| SW-G28 | SW-Gisloev/Glimminge | Sweden | autochthonous | 7,35 | 45 | 7,350 |  |  |  |
| SW-G29 | SW-Gisloev/Glimminge | Sweden | autochthonous | 5,54 | 43 | 5,778 |  |  |  |
| SW-G30 | SW-Gisloev/Glimminge | Sweden | autochthonous | 5,95 | 44 | 6,075 | AAC |  |  |
| SW-G31 | SW-Gisloev/Glimminge | Sweden | autochthonous | 6,08 | 45 | 6,080 | AAC | Allele 15,16,17 | 1/2 |
| SW-G32 | SW-Gisloev/Glimminge | Sweden | autochthonous | 6,47 | 44,5 | 6,537 | AAC |  |  |
| SW-G33 | SW-Gisloev/Glimminge | Sweden | autochthonous | 7,29 | 47 | 7,003 | AAC |  |  |
| SW-G34 | SW-Gisloev/Glimminge | Sweden | autochthonous | 6,92 | 46 | 6,781 |  |  |  |
| SW-G35 | SW-Gisloev/Glimminge | Sweden | autochthonous | 5,79 | 43 | 6,038 | AAC |  |  |
| SW-G36 | SW-Gisloev/Glimminge | Sweden | autochthonous | 6,76 | 46 | 6,624 | AAC |  |  |
| SW-G37 | SW-Gisloev/Glimminge | Sweden | autochthonous | 6,58 | 46 | 6,448 | AAC |  |  |
| SW-G38 | SW-Gisloev/Glimminge | Sweden | autochthonous | 6,92 | 44,5 | 6,992 | AAC-A |  |  |
| SW-G39 | SW-Gisloev/Glimminge | Sweden | autochthonous | 5,24 | 46 | 5,135 |  |  |  |
| SW-G40 | SW-Gisloev/Glimminge | Sweden | autochthonous | 6,33 | 45,5 | 6,266 | AAC | Allele 16,18,19 | 1/2 |
| SW-G41 | SW-Gisloev/Glimminge | Sweden | autochthonous | 5,42 | 44,5 | 5,476 | AAC |  |  |
| SW-G42 | SW-Gisloev/Glimminge | Sweden | autochthonous | 7,25 | 46,5 | 7,034 | AAC |  |  |
| SW-G43 | SW-Gisloev/Glimminge | Sweden | autochthonous | 7,56 | 41,5 | 8,147 | AAC |  |  |
| SW-G44 | SW-Gisloev/Glimminge | Sweden | autochthonous | 8,1 | 47 | 7,781 | AAC | Allele 15,18,19 | 1/1 |
| SW-G45 | SW-Gisloev/Glimminge | Sweden | autochthonous | 7,03 | 44,5 | 7,103 |  |  |  |
| SW-G46 | SW-Gisloev/Glimminge | Sweden | autochthonous | 4,28 | 38 | 5,003 | AAC |  |  |
| SW-G47 | SW-Gisloev/Glimminge | Sweden | autochthonous | 6,52 | 45,5 | 6,454 | AAC |  |  |
| SW-G48 | SW-Gisloev/Glimminge | Sweden | autochthonous | 5,85 | 43,5 | 6,036 | AAC | Allele 01,13,15,16,18,19 | 2/1 |
| SW-G49 | SW-Gisloev/Glimminge | Sweden | autochthonous | 7,59 | 46,5 | 7,364 | AAC |  |  |
| SW-G50 | SW-Gisloev/Glimminge | Sweden | autochthonous | 6,38 | 46 | 6,252 |  |  |  |
| SW-G51 | SW-Gisloev/Glimminge | Sweden | autochthonous | 6,35 | 44 | 6,483 | AAC |  |  |
| SW-G52 | SW-Gisloev/Glimminge | Sweden | autochthonous | 6,98 | 45 | 6,980 | AAC |  |  |
| SW-G53 | SW-Gisloev/Glimminge | Sweden | autochthonous | 5,81 | 44,5 | 5,870 | AAC-A | Allele 16,18,19 | 1/2 |
| SW-G54 | SW-Gisloev/Glimminge | Sweden | autochthonous | 3,89 | 39 | 4,440 |  |  |  |
| SW-G55 | SW-Gisloev/Glimminge | Sweden | autochthonous | 6,05 | 47 | 5,812 | AAC |  |  |
| SW-G56 | SW-Moelle | Sweden | allochthonous | 6,38 | 43 | 6,654 | DEG |  |  |
| SW-G57 | SW-Moelle | Sweden | allochthonous | 5,66 | 46 | 5,546 | DEG |  |  |
| SW-G58 | SW-Moelle | Sweden | allochthonous | 8,21 | 50 | 7,449 | DEG |  |  |
| SW-G59 | SW-Moelle | Sweden | allochthonous | 6,87 | 45 | 6,870 | DEG |  |  |
| SW-G60 | SW-Moelle | Sweden | allochthonous | 6,87 | 45 | 6,870 | DEG |  |  |
| SW-G61 | SW-Moelle | Sweden | allochthonous | 7,35 | 48 | 6,925 | DEG |  |  |
| SW-G62 | SW-Moelle | Sweden | allochthonous | 7,12 | 45,5 | 7,048 | AAC |  |  |
| SW-G63 | SW-Moelle | Sweden | allochthonous | 8,48 | 50 | 7,694 | DEG |  |  |
| SW-G64 | SW-Moelle | Sweden | allochthonous | 7,99 | 51 | 7,118 | DEG |  |  |
| SW-G65 | SW-Moelle | Sweden | allochthonous | 6,78 | 45 | 6,780 | DEG |  |  |
| SW-G66 | SW-Moelle | Sweden | allochthonous | 7,83 | 46 | 7,673 | DEG |  |  |
| SW-G67 | SW-Moelle | Sweden | allochthonous | 7,56 | 46 | 7,408 | DEG |  |  |
| SW-G68 | SW-Moelle | Sweden | allochthonous | 7,64 | 49 | 7,062 | DEG |  |  |
| SW-G69 | SW-Moelle | Sweden | allochthonous | 6,4 | 44 | 6,534 | DEG |  |  |
| SW-G70 | SW-Moelle | Sweden | allochthonous | 7,79 | 47 | 7,483 |  |  |  |
| SW-G71 | SW-Moelle | Sweden | allochthonous | 7,2 | 48 | 6,783 | DEG |  |  |
| SW-G72 | SW-Moelle | Sweden | allochthonous | 1,87 | 30 | 2,719 |  |  |  |
| SW-G73 | SW-Moelle | Sweden | allochthonous | 7,75 | 43 | 8,082 |  | Allele 01,03,05,10,13,15,16,18,19 | 1/2 |
| SW-G74 | SW-Moelle | Sweden | allochthonous | 6,62 | 43 | 6,904 | DEG |  | 1/2 |
| SW-G75 | SW-Moelle | Sweden | allochthonous | 3,08 | 34 | 3,990 | DEG |  |  |
| SW-G76 | SW-Moelle | Sweden | allochthonous | 4,5 | 40 | 5,017 | DEG |  |  |
| SW-G77 | SW-Foetene | Sweden | allochthonous | 10,36 | 47 | 9,952 | AAC |  |  |
| SW-G78 | SW-Foetene | Sweden | allochthonous | 10,69 | 47 | 10,269 | DEG |  |  |
| SW-G79 | SW-Foetene | Sweden | allochthonous | 10,92 | 47 | 10,490 | DEG | Allele 05,13,15,16,17,18,19 | 1/2 |
| SW-G80 | SW-Foetene | Sweden | allochthonous | 4,75 | 39 | 5,421 | DEG |  |  |
| SW-G81 | SW-Foetene | Sweden | allochthonous | 8,94 | 46 | 8,760 | DEG |  |  |
| SW-G82 | SW-Foetene | Sweden | allochthonous | 11,4 | 48 | 10,740 | DEG |  |  |
| SW-G83 | SW-Foetene | Sweden | allochthonous | 10,58 | 46 | 10,367 | AAC-A | Allele 09,11,13,15,17,18,19 | 1/1 |
| SW-G84 | SW-Foetene | Sweden | allochthonous | 11,91 | 51 | 10,610 | AAC |  |  |
| SW-G85 | SW-Foetene | Sweden | allochthonous | 11,98 | 48 | 11,287 | DEG | Allele 16,17,18,19 | 1/1 |
| SW-G86 | SW-Foetene | Sweden | allochthonous | 10,36 | 48 | 9,760 |  | Allele 04,09,19 | 2/2 |
| SW-G87 | SW-Foetene | Sweden | allochthonous | 10,16 | 46 | 9,956 | DEG | Allele 09,11,17,18 | 1/1 |
| SW-G88 | SW-Foetene | Sweden | allochthonous | 7,1 | 47 | 6,820 | DEG |  |  |
| SW-G89 | SW-Foetene | Sweden | allochthonous | 9,27 | 47 | 8,905 |  |  |  |
| SH-G01 | GE-Dannau | Germany | allochthonous | 8,74 | 50,5 | 7,857 | DEG |  |  |
| SH-G02 | GE-Dannau | Germany | allochthonous | 9 | 47 | 8,646 | DEG |  |  |
| SH-G03 | GE-Dannau | Germany | allochthonous | 8,8 | 46 | 8,623 | DEG |  |  |
| SH-G04 | GE-Dannau | Germany | allochthonous | 6,57 | 44,5 | 6,638 | DEG |  |  |
| SH-G05 | GE-Dannau | Germany | allochthonous |  |  |  |  | Allele 03,04,10,16,18,19 | 1/1 |
| SH-G06 | GE-Dannau | Germany | allochthonous | 8 | 45,5 | 7,919 | AAB | Allele 04,16,18,19 | 1/1 |
| SH-G07 | GE-Dannau | Germany | allochthonous | 9,76 | 50 | 8,855 | AAC |  |  |
| SH-G08 | GE-Dannau | Germany | allochthonous | 7,83 | 44,5 | 7,911 | AAC | Allele 15,16,18,19 | 1/1 |
| SH-G09 | GE-Dannau | Germany | allochthonous | 5,36 | 43 | 5,590 | AAB |  |  |
| SH-G10 | GE-Dannau | Germany | allochthonous | 9,09 | 46,5 | 8,819 | AAB |  |  |
| SH-G11 | GE-Dannau | Germany | allochthonous | 7,6 | 44 | 7,759 | AAB |  |  |
| SH-G12 | GE-Dannau | Germany | allochthonous |  |  |  | Excluded due to low sequencing coverage and quality | | |
| SH-G13 | GE-Dannau | Germany | allochthonous |  |  |  | AAC | Allele 16,18,19 | 1/1 |
| SH-G14 | GE-Dannau | Germany | allochthonous |  |  |  | AAC |  |  |
| SH-G15 | GE-Dannau | Germany | allochthonous |  |  |  | Excluded due to low sequencing coverage and quality | | |
| SH-G16 | GE-Dannau | Germany | allochthonous |  |  |  | AAC |  |  |
| SH-G17 | GE-Dannau | Germany | allochthonous |  |  |  | AAC |  |  |
| SH-G18 | GE-Dannau | Germany | allochthonous | 5,71 | 42 | 2,485 | AAB |  |  |
| SH-G19 | GE-Dannau | Germany | allochthonous | 5,54 | 42,5 | 2,442 | AAB |  |  |
| SH-G20 | GE-Dannau | Germany | allochthonous | 6,06 | 43 | 2,565 | AAB |  |  |
| SH-G21 | GE-Dannau | Germany | allochthonous | 6,59 | 44,5 | 2,526 | AAB |  |  |
| SH-G22 | GE-Dannau | Germany | allochthonous | 9,79 | 46 | 2,565 | AAB |  |  |
| SH-G23 | GE-Fehmarn | Germany | autochthonous | 9,61 | 49 | 2,565 | AAB | Allele 06,13,15 |  |
| SH-G24 | GE-Fehmarn | Germany | autochthonous | 10,35 | 50 | 2,639 | AAB | Allele 15,18,19 |  |
| SH-G25 | GE-Fehmarn | Germany | autochthonous | 13,31 | 50 | 12,076 | AAB | Allele 10,13,15,18,19 |  |
| SH-G26 | GE-Fehmarn | Germany | autochthonous | 10,4 | 45 | 10,400 | AAB | Allele 13,15,18,19 |  |
| SH-G27 | GE-Fehmarn | Germany | autochthonous | 11,74 | 53 | 10,093 | AAB | Allele 05,10,13,15,18,19 |  |
| SH-G28 | GE-Fehmarn | Germany | autochthonous | 8 | 45 | 8,000 | AAB | Allele 12 |  |
| SH-G29 | GE-Fehmarn | Germany | autochthonous | 8,72 | 47,5 | 8,295 | AAB | Allele 01,05,10,15,18,19 |  |
| SH-G30 | GE-Fehmarn | Germany | autochthonous | 8,41 | 47 | 8,079 | AAB |  |  |
| SH-G31 | GE-Fehmarn | Germany | autochthonous | 9,47 | 49 | 8,754 | AAB |  |  |
| SH-G32 | GE-Fehmarn | Germany | autochthonous | 9,37 | 44 | 9,567 |  |  |  |
| SH-G33 | GE-Fehmarn | Germany | autochthonous | 7,49 | 41 | 8,163 | AAB |  |  |
| SH-G34 | GE-Fehmarn | Germany | autochthonous | 9,1 | 42,5 | 9,593 | AAB |  |  |
| SH-G35 | GE-Fehmarn | Germany | autochthonous | 13,64 | 49 | 12,608 |  |  |  |
| SH-G36 | GE-Fehmarn | Germany | autochthonous | 13,7 | 48 | 12,907 |  |  |  |
| SH-G37 | GE-Fehmarn | Germany | autochthonous | 10,85 | 45 | 10,850 |  |  |  |
| SH-G38 | GE-Fehmarn | Germany | autochthonous | 9,34 | 44 | 9,536 |  |  |  |
| SH-G39 | GE-Fehmarn | Germany | autochthonous | 13,22 | 44,5 | 13,357 |  |  |  |
| SH-G40 | GE-Fehmarn | Germany | autochthonous | 14,87 | 47 | 14,285 |  |  |  |
| SH-G41 | GE-Fehmarn | Germany | autochthonous | 17,21 | 50 | 15,614 |  |  |  |
| SH-G42 | GE-Fehmarn | Germany | autochthonous | 15,58 | 49,5 | 14,267 |  |  |  |
| SH-G43 | GE-Hoegsdorf | Germany | allochthonous | 9,81 | 48 | 9,242 | AAB | Allele 03,14,16,17,18,19 | 1/1 |
| SH-G44 | GE-Hoegsdorf | Germany | allochthonous | 15,68 | 50 | 14,226 |  |  |  |
| SH-G45 | GE-Hoegsdorf | Germany | allochthonous | 11,38 | 43 | 11,868 |  |  |  |
| SH-G46 | GE-Hoegsdorf | Germany | allochthonous | 16,65 | 49 | 15,391 |  | Allele 13,15,16,17,19 | 1/1 |
| SH-G47 | GE-Hoegsdorf | Germany | allochthonous | 15,21 | 47 | 14,611 |  |  |  |
| SH-G48 | GE-Hoegsdorf | Germany | allochthonous | 14,89 | 46 | 14,591 |  |  |  |
| SH-G49 | GE-Hoegsdorf | Germany | allochthonous | 17,68 | 54 | 14,940 | DEG |  |  |
| SH-G50 | GE-Hoegsdorf | Germany | allochthonous | 10,55 | 47 | 10,135 | AAC | Allele 14,17,19 | 1/1 |
| SH-G51 | GE-Hoegsdorf | Germany | allochthonous | 5,32 | 42 | 5,670 | AAC | Allele 14,16,18,19 | 3/1 |
| SH-G52 | GE-Hoegsdorf | Germany | allochthonous | 8,61 | 48 | 8,112 | AAB | Allele 14,16,17,18,19 | 1/1 |
| SH-G53 | GE-Hoegsdorf | Germany | allochthonous | 15,09 | 48 | 14,217 |  | Allele 14,16,17,18,19 | 3/1 |
| SH-G54 | GE-Hoegsdorf | Germany | allochthonous | 10,55 | 46 | 10,338 |  |  |  |
| SH-G55 | GE-Hoegsdorf | Germany | allochthonous | 13,86 | 48 | 13,058 |  |  |  |
| SH-G56 | GE-Hoegsdorf | Germany | allochthonous | 13,79 | 50 | 12,511 |  |  |  |
| SH-G57 | GE-Hoegsdorf | Germany | allochthonous | 12,12 | 49 | 11,203 |  |  |  |
| SH-G58 | GE-Hoegsdorf | Germany | allochthonous | 5,65 | 43 | 5,892 | DEG |  |  |
| SH-G59 | GE-Hoegsdorf | Germany | allochthonous | 8,08 | 45 | 8,080 | DEG | Allele 14,17,18,19 | 1/1 |
| SH-G60 | GE-Hoegsdorf | Germany | allochthonous | 9,05 | 50 | 8,211 | DEG | Allele 14,16,17,19 | 1/1 |
| SH-G61 | GE-Hoegsdorf | Germany | allochthonous | 9,61 | 49,5 | 8,800 | DEG | Allele 15,16,17,18 | 3/1 |
| SH-G62 | GE-Hoegsdorf | Germany | allochthonous | 10,21 | 43 | 10,648 |  | Allele 14,18,19 | 1/1 |
| SH-G63 | GE-Hoegsdorf | Germany | allochthonous | 9,77 | 42 | 10,413 | DEG |  |  |
| SH-G64 | GE-Hoegsdorf | Germany | allochthonous | 5,98 | 43 | 6,236 | AAB |  |  |
| SH-G65 | GE-Eutin | Germany | allochthonous | 5,55 | 45,5 | 5,494 | DEG |  |  |
| SH-G66 | GE-Eutin | Germany | allochthonous | 11,53 | 50 | 10,461 | AAB | Allele 16,19 | 1/2 |
| SH-G67 | GE-Eutin | Germany | allochthonous | 11,12 | 51,5 | 9,817 | DEG |  |  |
| SH-G68 | GE-Eutin | Germany | allochthonous | 9,82 | 49 | 9,077 | DEG |  |  |
| SH-G69 | GE-Eutin | Germany | allochthonous | 12,9 | 44 | 13,171 |  | Allele 18 | 2/4 |
| SH-G70 | GE-Eutin | Germany | allochthonous | 12,39 | 49 | 11,453 |  |  |  |
| SH-G71 | GE-Eutin | Germany | allochthonous | 11,79 | 47 | 11,326 |  |  |  |
| SH-G72 | GE-Eutin | Germany | allochthonous | 15,37 | 53 | 13,214 |  |  |  |
| SH-G73 | GE-Eutin | Germany | allochthonous | 9,32 | 45 | 9,320 |  |  |  |
| SH-G74 | GE-Eutin | Germany | allochthonous | 13,29 | 51 | 11,839 |  |  |  |
| SH-G75 | GE-Testorf | Germany | allochthonous | 12 | 46 | 11,759 | AAB |  |  |
| SH-G76 | GE-Testorf | Germany | allochthonous | 12,5 | 41,5 | 13,471 | AAB |  |  |
| SH-G77 | GE-Testorf | Germany | allochthonous | 13 | 48 | 12,248 | DEG |  |  |
| SH-G78 | GE-Testorf | Germany | allochthonous | 13 | 58,5 | 10,202 | DEG |  |  |
| SH-G79 | GE-Testorf | Germany | allochthonous | 13 | 47 | 12,488 | AAB |  |  |
| SH-G80 | GE-Testorf | Germany | allochthonous | 14 | 43 | 14,600 | AAB | Allele 10,16,18,19 | 1/2 |
| SH-G81 | GE-Testorf | Germany | allochthonous | 13,5 | 44,5 | 13,640 | AAB |  |  |
| SH-G82 | GE-Testorf | Germany | allochthonous | 10,27 | 49 | 9,493 |  |  |  |
| SH-G83 | GE-Testorf | Germany | allochthonous | 8,76 | 43,5 | 9,039 |  |  |  |
| SH-G84 | GE-Testorf | Germany | allochthonous | 9,42 | 44 | 9,618 |  |  | 1/2 |
| SH-G85 | GE-Testorf | Germany | allochthonous | 13,5 | 43 | 14,079 | AAB |  |  |
| SH-G86 | GE-Testorf | Germany | allochthonous | 13 | 46 | 12,739 | AAB | Allele 04,19 | 2/2 |
| SH-G87 | GE-Testorf | Germany | allochthonous | 14 | 45 | 14,000 | AAB |  |  |
| SH-G88 | GE-Testorf | Germany | allochthonous | 13 | 47 | 12,488 | AAB |  |  |
| SH-G89 | GE-Testorf | Germany | allochthonous | 13 | 44 | 13,273 | AAB |  |  |
| SH-G90 | GE-Testorf | Germany | allochthonous | 11,51 | 46 | 11,279 |  |  |  |
| SH-G91 | GE-Testorf | Germany | allochthonous | 8,27 | 42 | 8,814 |  |  |  |
| SH-G92 | GE-Testorf | Germany | allochthonous | 9,05 | 43 | 9,438 |  |  |  |
| SH-G93 | GE-Testorf | Germany | allochthonous | 8,52 | 45 | 8,520 |  |  |  |
| SH-G94 | GE-Testorf | Germany | allochthonous | 10,62 | 46 | 10,407 |  |  |  |
| SH-G95 | GE-Testorf | Germany | allochthonous | 11,12 | 45 | 11,120 |  |  |  |
| SH-G96 | GE-Testorf | Germany | allochthonous | 8,62 | 41 | 9,394 |  |  |  |
| SH-G97 | GE-Testorf | Germany | allochthonous | 10,15 | 47 | 9,750 |  |  |  |
| SH-G98 | GE-Testorf | Germany | allochthonous | 9,86 | 46 | 9,662 |  |  |  |
| SH-G99 | GE-Testorf | Germany | allochthonous | 10,85 | 46,5 | 10,526 |  |  |  |
| SH-G100 | GE-Testorf | Germany | allochthonous | 8,25 | 45 | 8,250 |  |  |  |
| A-G1 | A-Wien-1 | Austria | autochthonous | 3,5 | 42,8 | 3,666 | AAB |  |  |
| A-G2 | A-Wien-1 | Austria | autochthonous | 5,5 | 40 | 6,132 | AAB | Allele 02,08,12,14,17 | 9/6 |
| A-G3 | A-Wien-1 | Austria | autochthonous | 6,5 | 45 | 6,500 | AAB |  |  |
| A-G4 | A-Wien-1 | Austria | autochthonous | 6,5 | 44 | 6,636 | AAB |  |  |
| A-G5 | A-Wien-1 | Austria | autochthonous | 7,5 | 45 | 7,500 |  |  |  |
| A-G6 | A-Wien-1 | Austria | autochthonous | 7,5 | 43,1 | 7,805 |  |  |  |
| A-G7 | A-Wien-1 | Austria | autochthonous | 7,5 | 45 | 7,500 | DEG |  |  |
| A-G8 | A-Wien-1 | Austria | autochthonous | 5,5 | 40 | 6,132 |  |  |  |
| A-G9 | A-Wien-1 | Austria | autochthonous | 6,5 | 44,2 | 6,609 | DEG |  |  |
| A-G10 | A-Wien-1 | Austria | autochthonous | 7,5 | 44,5 | 7,578 | DEG |  |  |
| A-G11 | A-Wien-2 | Austria | autochthonous | 5,5 | 40 | 6,132 | DEG |  |  |
| A-G12 | A-Wien-2 | Austria | autochthonous | 7,5 | 46,5 | 7,276 | DEG |  |  |
| A-G13 | A-Wien-2 | Austria | autochthonous | 8,5 | 45 | 8,500 | DEG |  |  |
| A-G14 | A-Wien-2 | Austria | autochthonous | 6,5 | 44 | 6,636 | DEG |  |  |
| A-G15 | A-Wien-2 | Austria | autochthonous | 8,5 | 42,5 | 8,961 | DEG |  |  |
| A-G16 | A-Wien-2 | Austria | autochthonous | 7,5 | 44 | 7,657 | DEG |  |  |
| A-G17 | A-Wien-2 | Austria | autochthonous | 9,5 | 47 | 9,126 | DEG |  |  |
| A-G18 | A-Wien-2 | Austria | autochthonous | 7,5 | 44,8 | 7,531 | DEG |  |  |
| A-G19 | A-Wien-2 | Austria | autochthonous | 6,5 | 40 | 7,247 | DEG |  |  |
| A-G20 | A-Wien-2 | Austria | autochthonous | 5,5 | 38,5 | 6,352 | DEG |  |  |
| A-G21 | A-Wien-2 | Austria | autochthonous | 3,5 | 34 | 4,534 | DEG |  |  |
| A-G22 | A-Wien-2 | Austria | autochthonous | 2,5 | 29 | 3,751 | DEG | Allele 06,08,13,14,17 | 2/2 |
| A-G23 | A-Wien-3 | Austria | autochthonous | 9,5 | 45,5 | 9,404 | AAB |  |  |
| A-G24 | A-Wien-3 | Austria | autochthonous | 9,5 | 41 | 10,353 |  | Allele 08,17 | 1/4 |
| A-G25 | A-Wien-3 | Austria | autochthonous | 7,5 | 37,5 | 8,876 | DEG |  |  |
| A-G26 | A-Wien-3 | Austria | autochthonous | 8,5 | 46 | 8,329 | DEG |  |  |
| A-G27 | A-Wien-3 | Austria | autochthonous | 7,5 | 43 | 7,822 |  |  |  |
| A-G28 | A-Wien-3 | Austria | autochthonous | 7,5 | 39 | 8,560 | DEG |  |  |
| A-G29 | A-Wien-3 | Austria | autochthonous | 7,5 | 41 | 8,173 | DEG |  |  |
| A-G30 | A-Wien-3 | Austria | autochthonous | 7,5 | 40 | 8,362 | DEG |  |  |
| A-G31 | A-Wien-3 | Austria | autochthonous | 8,5 | 39 | 9,701 | DEG |  |  |
| A-G32 | A-Wien-3 | Austria | autochthonous | 7,5 | 42 | 7,993 | DEG |  |  |
| A-G33 | A-Wien-3 | Austria | autochthonous | 6,5 | 37 | 7,788 | AAB |  |  |
| A-G34 | A-Wien-3 | Austria | autochthonous | 6,5 | 36 | 7,988 | DEG |  |  |
| A-G35 | A-Wien-3 | Austria | autochthonous | 7,5 | 40 | 8,362 | DEG |  |  |
| A-G36 | A-Wien-3 | Austria | autochthonous | 6,5 | 37,8 | 7,636 | DEG |  |  |
| A-G37 | A-Wien-4 | Austria | autochthonous | 5,5 | 34 | 7,125 | DEG |  |  |
| A-G38 | A-Wien-4 | Austria | autochthonous | 5,5 | 38 | 6,430 | DEG |  |  |
| A-G39 | A-Wien-4 | Austria | autochthonous | 6,5 | 37,5 | 7,692 | DEG | Allele 14,17 | 1/1 |
| A-G40 | A-Wien-4 | Austria | autochthonous | 6,5 | 40 | 7,247 | DEG |  |  |
| A-G41 | A-Wien-4 | Austria | autochthonous | 4,5 | 34,5 | 5,752 | DEG |  |  |
| A-G42 | A-Wien-4 | Austria | autochthonous | 5,5 | 34,5 | 7,030 | DEG |  |  |
| A-G43 | A-Wien-4 | Austria | autochthonous | 6,5 | 40,8 | 7,116 | DEG |  |  |
| A-G44 | A-Wien-5 | Austria | autochthonous | 7,5 | 44 | 7,657 | DEG |  |  |
| A-G45 | A-Wien-5 | Austria | autochthonous | 6,5 | 40 | 7,247 | AAB |  |  |
| A-G46 | A-Wien-5 | Austria | autochthonous | 7,5 | 44 | 7,657 | DEG |  |  |
| A-G47 | A-Wien-5 | Austria | autochthonous | 8,5 | 45,5 | 8,414 | DEG |  |  |
| A-G48 | A-Wien-5 | Austria | autochthonous | 6,5 | 38,5 | 7,507 | AAB |  | 1/1 |
| A-G49 | A-Wien-5 | Austria | autochthonous | 7,5 | 42 | 7,993 | DEG |  |  |
| A-G50 | A-Wien-5 | Austria | autochthonous | 6,5 | 39 | 7,418 | DEG |  |  |
| A-G51 | A-NNO von Hohenau an der March/Hrut-Sutte | Austria | autochthonous |  |  |  | DEG |  |  |
| A-G52 | A-NNO von Hohenau an der March/Hrut-Sutte | Austria | autochthonous |  |  |  | DEG | Allele 06,08,14,17 | 1/1 |
| A-G53 | A-NNO von Hohenau an der March/Hrut-Sutte | Austria | autochthonous |  |  |  | DEG |  |  |
| A-G54 | A-NNO von Hohenau an der March/Hrut-Sutte | Austria | autochthonous |  |  |  | DEG |  |  |
| A-G55 | A-NNO von Hohenau an der March/Hrut-Sutte | Austria | autochthonous |  |  |  | DEG |  |  |
| A-G56 | A-NNO von Hohenau an der March/Hrut-Sutte | Austria | autochthonous |  |  |  | DEG-T | Allele 02,10,11,14,15,16,17,19 | 1/1 |
| A-G57 | A-NNO von Hohenau an der March/Hrut-Sutte | Austria | autochthonous |  |  |  | DEG |  |  |
| A-G58 | A-NNO von Hohenau an der March/Hrut-Sutte | Austria | autochthonous |  |  |  | DEG |  |  |
| A-G60 | A-NNO von Hohenau an der March/Hrut-Sutte | Austria | autochthonous |  |  |  | Excluded due to low sequencing coverage and quality | | |
| A-G61 | A-Zicklacken, Neusiedler See | Austria | autochthonous | 4 | 31,33 | 5,589 | DEG-T |  |  |
| A-G62 | A-Zicklacken, Neusiedler See | Austria | autochthonous | 3 | 32,83 | 4,014 | DEG |  |  |
| A-G63 | A-Zicklacken, Neusiedler See | Austria | autochthonous | 2 | 30,95 | 2,826 | DEG | Allele 02,12,14,17 | 1/5 |
| A-G64 | A-Zicklacken, Neusiedler See | Austria | autochthonous | 2 | 30,45 | 2,869 | DEG-T |  |  |
| A-G65 | A-Zicklacken, Neusiedler See | Austria | autochthonous | 2 | 31,7 | 2,764 | DEG |  | 1/1 |
| A-G66 | A-Zicklacken, Neusiedler See | Austria | autochthonous | 2 | 28,47 | 3,053 | DEG |  |  |
| A-G67 | A-Zicklacken, Neusiedler See | Austria | autochthonous | 2 | 26,03 | 3,316 | DEG |  |  |
| A-G68 | A-Zicklacken, Neusiedler See | Austria | autochthonous | 2 | 24,04 | 3,569 | DEG |  |  |
| A-G69 | A-Zicklacken, Neusiedler See | Austria | autochthonous | 1,5 | 25,22 | 2,561 |  |  |  |
| A-G70 | A-Zicklacken, Neusiedler See | Austria | autochthonous | 2 | 22,31 | 3,824 | DEG |  |  |
| A-G71 | A-Weiden, Strassengraben, Neusiedler See | Austria | autochthonous | 6 | 41,87 | 6,413 | DEG |  |  |
| A-G72 | A-Weiden, Strassengraben, Neusiedler See | Austria | autochthonous | 6 | 43,81 | 6,150 |  |  |  |
| A-G73 | A-Weiden, Strassengraben, Neusiedler See | Austria | autochthonous | 5 | 43,52 | 5,157 | DEG |  |  |
| A-G74 | A-Weiden, Strassengraben, Neusiedler See | Austria | autochthonous | 4 | 46,26 | 3,899 | DEG | Allele 17 | 1/8 |
| A-G75 | A-Weiden, Strassengraben, Neusiedler See | Austria | autochthonous | 5 | 37,02 | 5,988 | DEG |  |  |
| A-G76 | A-Weiden, Strassengraben, Neusiedler See | Austria | autochthonous | 7 | 43,81 | 7,175 | DEG |  |  |
| A-G77 | A-Weiden, Strassengraben, Neusiedler See | Austria | autochthonous | 5 | 39,56 | 5,632 |  | Allele 12 | 2/2 |
| A-G78 | A-Weiden, Strassengraben, Neusiedler See | Austria | autochthonous | 6 | 43,4 | 6,204 | DEG |  |  |
| A-G79 | A-Weiden, Strassengraben, Neusiedler See | Austria | autochthonous | 6 | 43,74 | 6,159 | DEG |  |  |
| A-G80 | A-Weiden, Strassengraben, Neusiedler See | Austria | autochthonous | 2 | 34,39 | 2,564 | DEG |  |  |
| A-G81 | A-Weiden, Strassengraben, Neusiedler See | Austria | autochthonous | 4,15 | 36,73 | 5,006 | DEG |  |  |
| A-G82 | A-Zitzmanndorfer Wiesen, Neusiedler See | Austria | autochthonous | 1,53 | 30,49 | 2,192 | DEG |  |  |
| A-G83 | A-Zitzmanndorfer Wiesen, Neusiedler See | Austria | autochthonous | 4,47 | 37,87 | 5,242 | DEG-A | Allele 12,17 | 1/1 |
| A-G84 | A-Zitzmanndorfer Wiesen, Neusiedler See | Austria | autochthonous | 5,32 | 42,93 | 5,557 | DEG |  |  |
| A-G85 | A-Zitzmanndorfer Wiesen, Neusiedler See | Austria | autochthonous | 2,15 | 30,84 | 3,048 | DEG |  |  |
| A-G86 | A-Zitzmanndorfer Wiesen, Neusiedler See | Austria | autochthonous | 1,77 | 28,56 | 2,694 | DEG | Allele 09,11,17 | 1/7 |
| A-G87 | A-Zitzmanndorfer Wiesen, Neusiedler See | Austria | autochthonous | 2,03 | 31,24 | 2,844 | DEG |  |  |
| A-G88 | A-Zitzmanndorfer Wiesen, Neusiedler See | Austria | autochthonous | 1,74 | 31,97 | 2,386 | DEG |  |  |
| A-G89 | A-Zitzmanndorfer Wiesen, Neusiedler See | Austria | autochthonous | 2,67 | 33,17 | 3,539 | DEG |  |  |
| A-G90 | A-Zitzmanndorfer Wiesen, Neusiedler See | Austria | autochthonous | 1,71 | 28,71 | 2,590 | DEG |  |  |
| A-G91 | A-Hansag, Neusiedler See | Austria | autochthonous | 6,77 | 43,19 | 7,032 | DEG |  |  |
| A-G92 | A-Hansag, Neusiedler See | Austria | autochthonous | 8,2 | 46,62 | 7,936 |  |  |  |
| A-G93 | A-Hansag, Neusiedler See | Austria | autochthonous | 7,13 | 41,37 | 7,706 | DEG |  |  |
| A-G94 | A-Hansag, Neusiedler See | Austria | autochthonous | 8,06 | 46,97 | 7,747 | DEG |  |  |
| A-G95 | A-Hansag, Neusiedler See | Austria | autochthonous | 4,91 | 40,7 | 5,387 | DEG-T |  |  |
| A-G96 | A-Hansag, Neusiedler See | Austria | autochthonous | 6,24 | 43,25 | 6,473 | DEG | Allele 12,14,17,18 | 1/1 |
| A-G97 | A-Hansag, Neusiedler See | Austria | autochthonous | 7,55 | 45,41 | 7,487 | DEG |  |  |
| A-G98 | A-Hansag, Neusiedler See | Austria | autochthonous | 7,83 | 46,59 | 7,583 | DEG |  |  |
| A-G99 | A-Hansag, Neusiedler See | Austria | autochthonous | 8,33 | 48,35 | 7,795 | DEG | Allele 12,17 | 1/1 |
| A-G100 | A-Hansag, Neusiedler See | Austria | autochthonous | 7,73 | 45,69 | 7,622 | DEG-T |  |  |
| A-G101 | A-Wilfleinsdorf, Bruck an der Leitha | Austria | autochthonous |  |  |  | DEG |  |  |
| A-G102 | A-Wilfleinsdorf, Bruck an der Leitha | Austria | autochthonous |  |  |  | DEG |  |  |
| A-G103 | A-Wilfleinsdorf, Bruck an der Leitha | Austria | autochthonous |  |  |  | DEG |  |  |
| A-G104 | A-Wilfleinsdorf, Bruck an der Leitha | Austria | autochthonous |  |  |  | DEG |  |  |
| A-G105 | A-Wilfleinsdorf, Bruck an der Leitha | Austria | autochthonous |  |  |  |  | Allele 14,17 | 1/1 |
| A-G106 | A-Wilfleinsdorf, Bruck an der Leitha | Austria | autochthonous |  |  |  | DEG |  |  |
| A-G107 | A-Wilfleinsdorf, Bruck an der Leitha | Austria | autochthonous |  |  |  | DEG |  |  |
| A-G108 | A-Wilfleinsdorf, Bruck an der Leitha | Austria | autochthonous |  |  |  | DEG |  |  |
| A-G109 | A-Wilfleinsdorf, Bruck an der Leitha | Austria | autochthonous |  |  |  | DEG |  |  |
| A-G110 | A-Wilfleinsdorf, Bruck an der Leitha | Austria | autochthonous |  |  |  | Excluded due to low sequencing coverage and quality | | |
| NS_A1 | Neusiedler See | Austria | autochthonous |  |  |  |  | Allele 11,14,17 | 1/2 |
| NS_A2 | Neusiedler See | Austria | autochthonous |  |  |  |  |  | 3/2 |
| NS_A3 | Neusiedler See | Austria | autochthonous |  |  |  |  | Allele 14,17 | 1/1 |

**Table A7:** ANOVA test results for mean SMI comparison between Austrian autochthonous and northern autochthonous and allochthonous populations (German and Swedish combined). Asterisk (*) indicates statistical significance after a sequential Bonferroni correction (*P* = 0.05). AU – Austria, NORTH all. – introgressed Northern Germany and Southern Sweden, NORTH aut. – non-introgressed Northern Germany and Southern Sweden.

|  | **AU** | **NORTH all.** |
| --- | --- | --- |
| **NORTH all.** | **3.5e-12***** | - |
| **NORTH aut.** | **0.00031**** | **0.02515*** |

**Table A8:** ANOVA test results for mean SMI comparison between Austrian autochthonous, German and Swedish autochthonous and allochthonous populations. Asterisk (*) indicates statistical significance after a sequential Bonferroni correction (*P* = 0.05). Austrian populations (Au), German introgressed (Ge-all.), German non-introgressed (Ge-aut.), Swedish introgressed (Sw-all.), and Swedish non-introgressed (Sw-aut.).

| SMI | **Au** | **Ge-all.** | **Ge-aut.** | **SW-all.** |
| --- | --- | --- | --- | --- |
| **Ge-all.** | **< 2e-16***** | - | - | - |
| **Ge-aut.** | **1.4e-14***** | **0.691** | - | - |
| **SW-all.** | **0.077** | **1.2e-13***** | **2.3e-09***** | - |
| **SW-aut.** | **0.890** | **1.6e-15***** | **1.7e-11***** | **0.259** |

| Body length | **Au** | **Ge-all.** | **Ge-aut.** | **SW-all.** |
| --- | --- | --- | --- | --- |
| **Ge-all.** | **< 2e-16***** | - | - | - |
| **Ge-aut.** | **1.4e-10***** | **1** | - | - |
| **SW-all.** | **1.9e-09***** | **0.072** | **0.096** | - |
| **SW-aut.** | **2.9e-08***** | **0.268** | **0.268** | **1** |

| Body mass | **Au** | **Ge-all.** | **Ge-aut.** | **SW-all.** |
| --- | --- | --- | --- | --- |
| **Ge-all.** | **< 2e-16***** | - | - | - |
| **Ge-aut.** | **< 2e-16***** | **0.417** | - | - |
| **SW-all..** | **0.002**** | **6.7e-13***** | **2.6e-09***** | - |
| **SW-aut.** | **0.417** | **1.8e-14***** | **3.6e-11***** | **0.343** |

**Figures**

**Fig. A1:** Haplotype frequencies of mitochondrial control region (d-loop) for all sampling sites for *B. bombina* (n=297).

**Fig. A2:** (a) Comparison of body mass for 272 *B. bombina* individuals, between Austrian, northern (German and Swedish) introgressed (NORTH-all.) and northern non-introgressed (NORTH-aut.) populations. (b) Comparison of body length (Snout-Vent-Length) for 272 *B. bombina* individuals, between Austrian, northern (German and Swedish) introgressed (NORTH-all.) and northern non-introgressed (NORTH-aut.) populations. The horizontal black bars represent median values and whiskers refer to the minimum and maximum values observed within the data for each population. Potential outliers are shown as outlined circles.

**
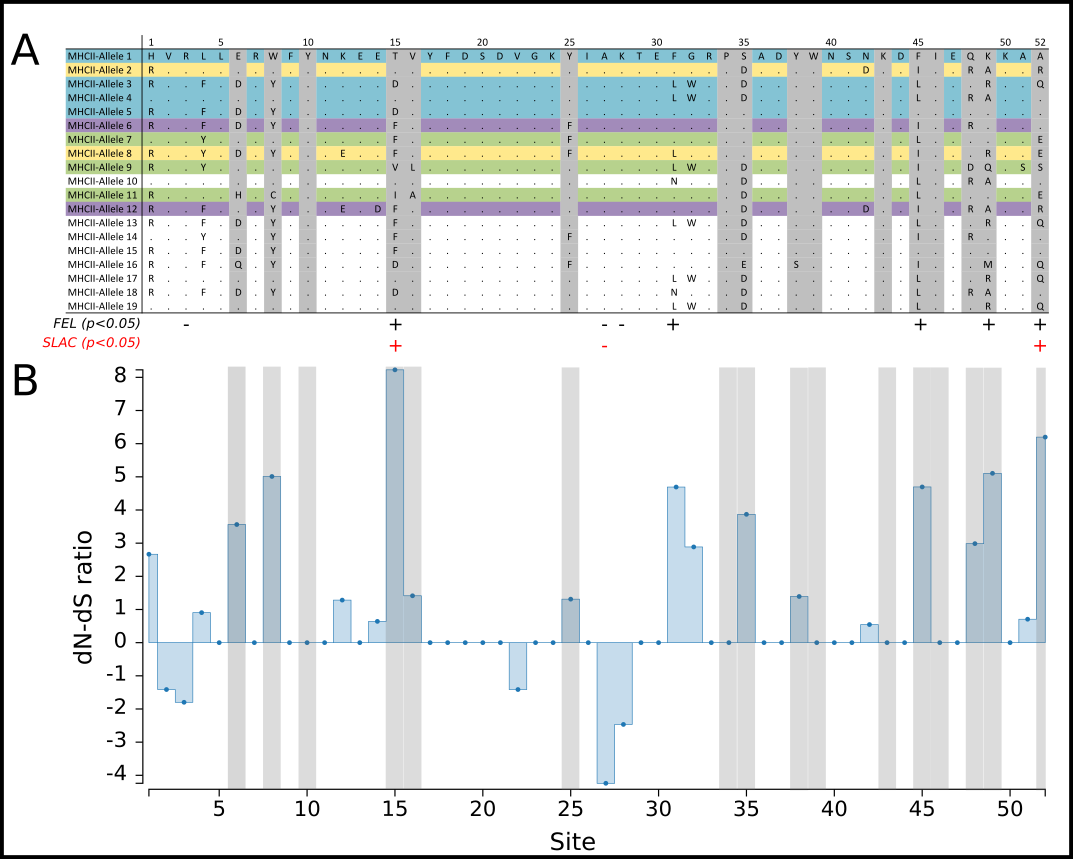
**

**Fig. A3:** (a) Amino acid alignment of MHC II exon 2 alleles detected in this study for *B. bombina***.** Putative antigen binding sites (ABS) known from human leukocyte antigen genes (HLA), which are part of the the major histocompatibility complex (MHC) gene complex are shaded in grey (Brown et al., 1993; Tong et al., 2006). Plus sign (+) marks codons under positive/diversifying selection, (-) marks codons under negative/purifying selection as inferred by a Fixed effects likelihood (FEL) and Single-Likelihood Ancestor (SLAC) test (p = p-value) (b) Ratio of the number of nonsynonymous substitutions per nonsynonymous site to the number of synonymous substitutions per synonymous site (dN/dS) graph from the SLAC analysis for each amino acid alignment position. Bars in alignment with (A)

**

**Fig. A4:** Scaled Mass Index (SMI) in relation to number of MHC II exon 2 alleles per individual (allele count).

**Fig. A5:** Bar plot of all individual SMIs and MHCII exon 2 allele counts for each individual respectively. SW-Sweden, AU-Austria, GE-Germany.

**
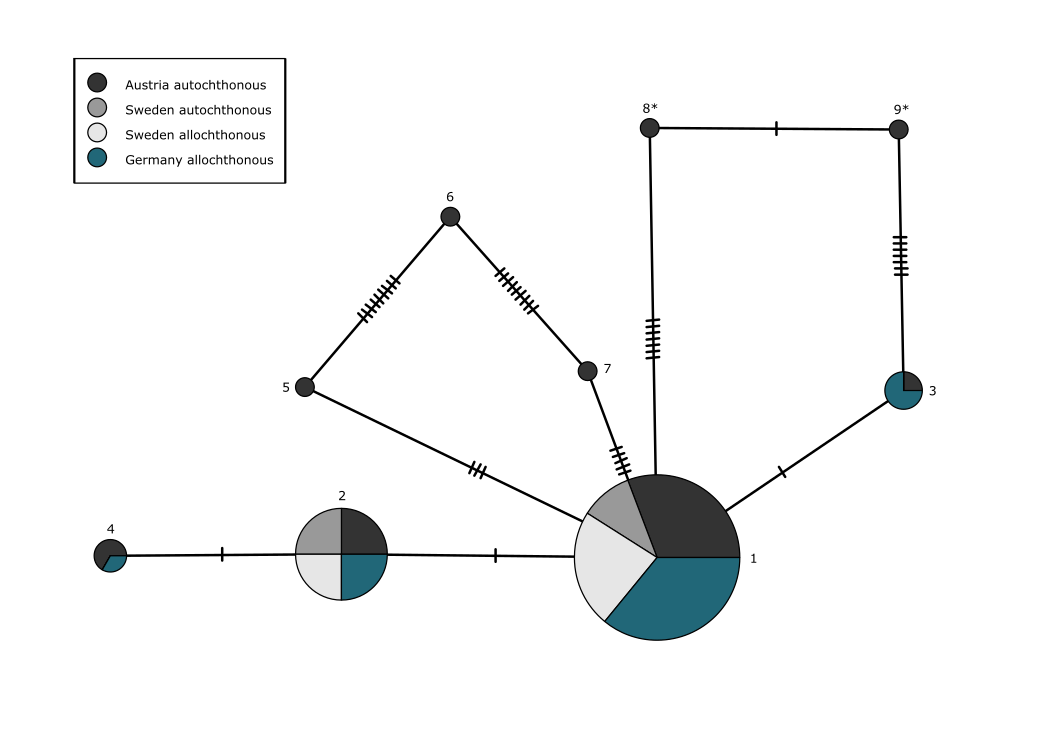
**

**Fig. A6:** Allele network of the HSP70 kDa gene for all introgressed (allochthonous) and non-introgressed (autochthonous) *B. bombina* populations*.* Alleles containing nonsynonymous changes in the amino acid sequence are indicated by an asterisk.
